# The C-Terminal Domains SnRK2 Box and ABA Box Have a Role in Sugarcane SnRK2s Auto-Activation and Activity

**DOI:** 10.3389/fpls.2019.01105

**Published:** 2019-09-17

**Authors:** Germanna Lima Righetto, Dev Sriranganadane, Levon Halabelian, Carla G. Chiodi, Jonathan M. Elkins, Katlin B. Massirer, Opher Gileadi, Marcelo Menossi, Rafael M. Couñago

**Affiliations:** ^1^Functional Genome Laboratory, Department of Genetics, Evolution, and Bioagents, Institute of Biology, State University of Campinas, Campinas, Brazil; ^2^Centro de Química Medicinal (CQMED), Centro de Biologia Molecular e Engenharia Genética (CBMEG), Universidade Estadual de Campinas (UNICAMP), Campinas, Brazil; ^3^Structural Genomics Consortium, Departamento de Genética e Evolução, Instituto de Biologia, UNICAMP, Campinas, Brazil; ^4^Structural Genomics Consortium, MaRS Centre, Toronto, ON, Canada; ^5^Structural Genomics Consortium, Nuffield Department of Medicine, University of Oxford, Oxford, United Kingdom

**Keywords:** abscisic acid, abiotic stress, SnRK2, crop plant, kinase regulation, sugarcane

## Abstract

Resistance to drought stress is fundamental to plant survival and development. Abscisic acid (ABA) is one of the major hormones involved in different types of abiotic and biotic stress responses. ABA intracellular signaling has been extensively explored in *Arabidopsis thaliana* and occurs *via* a phosphorylation cascade mediated by three related protein kinases, denominated SnRK2s (SNF1-related protein kinases). However, the role of ABA signaling and the biochemistry of SnRK2 in crop plants remains underexplored. Considering the importance of the ABA hormone in abiotic stress tolerance, here we investigated the regulatory mechanism of sugarcane SnRK2s—known as stress/ABA-activated protein kinases (SAPKs). The crystal structure of ScSAPK10 revealed the characteristic SnRK2 family architecture, in which the regulatory SnRK2 box interacts with the kinase domain αC helix. To study sugarcane SnRK2 regulation, we produced a series of mutants for the protein regulatory domains SnRK2 box and ABA box. Mutations in ScSAPK8 SnRK2 box aimed at perturbing its interaction with the protein kinase domain reduced protein kinase activity *in vitro*. On the other hand, mutations to ScSAPK ABA box did not impact protein kinase activity but did alter the protein autophosphorylation pattern. Taken together, our results demonstrate that both SnRK2 and ABA boxes might play a role in sugarcane SnRK2 function.

## Introduction

The phytohormone abscisic acid (ABA) is a central regulator of plant responses to abiotic stress. ABA triggers protective plant responses leading to stomatal closure, seed dormancy, inhibition of growth, and germination (Mustilli, 2002; Yoshida et al., 2002; Yoshida et al., 2006; Fujii et al., 2007; Fujii and Zhu, 2009). ABA’s signaling role is carried out by a protein phosphorylation cascade that depends on the interplay between the activities of SnRK2 kinases and protein phosphatase 2C (PP2C) (Fujii et al., 2009; Umezawa et al., 2010).

In eudicotyledons, members of the SnRK2 sub-family of serine-threonine kinases (SnRK2.2/2.3/2.6 in Arabidopsis) are the positive regulators of ABA signaling and activate downstream transcription factors, leading to the expression of stress-responsive genes (Fujii and Zhu, 2009; Fujii et al., 2009; Fujita et al., 2009; Nakashima et al., 2009). Counterpart kinases in monocots are known as stress/ABA-activated protein kinases (SAPK8/9/10) (Kobayashi et al., 2004; Yoshida et al., 2006). ABA-responsive kinases from both mono- and eudicotyledons are expected to have a conserved modular architecture and to be involved in environmental sensing and stress response (Kulik et al., 2011).

The C-terminal SnRK2 box is essential for kinase activation by hyperosmotic stress and displays high-sequence conservation among members of the SnRK2 subfamily (Kobayashi et al., 2004; Yoshida et al., 2006). The crystallographic structures of Arabidopsis SnRK2.3 and 2.6 have shown that the SnRK2 box folds into a helix and packs against the catalytically important αC helix within the protein kinase domain (Ng et al., 2011; Yunta et al., 2011). Mutational studies have demonstrated that the interaction between these two helices is crucial for kinase autoactivation and subsequent phosphorylation of the transcription factor ABF2 (Ng et al., 2011).

SnRK2/SAPK activity is modulated by direct interaction with PP2C phosphatases, which, in turn, depends on intracellular ABA levels. In the presence of the hormone, the phosphatase activity is impaired by the interaction with the complex formed by ABA and PYL/PYR/RCAR receptors (Ma et al., 2009; Melcher et al., 2009; Miyazono et al., 2009; Nakashima et al., 2009; Park et al., 2009; Umezawa et al., 2009; Yin et al., 2009; Moreno-Alvero et al., 2017). The complex blocks PP2C substrate entry and prevents SnRK2 inactivation by dephosphorylation. In the absence of ABA, PP2C is released from the complex with PYL/PYR/RCAR receptors and can interact with the SnRK2 kinases, leading to kinase dephosphorylation and repression of ABA-response. The interaction between kinase and phosphatase is mediated by another C-terminal motif, known as ABA box, only preserved in the ABA-responsive members of the SnRK2 subfamily (Umezawa et al., 2009; Vlad et al., 2009; Soon et al., 2012).

SnRK2 subfamily members have been identified in several crop plants, such as rice, maize, cotton, and wheat (Kobayashi et al., 2004; Huai et al., 2008; Zhang et al., 2010; Liu et al., 2017). Just like their counterparts from Arabidopsis, these proteins have been shown to mediate plant responses to abiotic stress and ABA. In *Saccharum officinarum* L. (So) sugarcane, a recent study identified ten SnRK2 subfamily members, three of which (SoSAPK8/9/10) have the characteristic ABA box in their C-terminus and, accordingly, are responsive to ABA (Li et al., 2017). Despite these studies, currently, there is no structural and biochemical information on SnRK2 subfamily members from crop plants. Moreover, the role of the regulatory domains SnRK2 box and ABA box in protein activity and activation remain unclear for sugarcane and other crop plants, in contrast with the extensive characterization in Arabidopsis.

In this study, we report the crystal structure of SAPK10 from the crop plant sugarcane (*Saccharum* ssp. hybrids). We also investigated how SnRK2 and ABA boxes modulate the activity of SAPK8/9/10. These analyses confirmed that, overall, the SnRK2 box within sugarcane SAPKs preserves its role in protein activity, albeit to a lesser extent when compared to the Arabidopsis proteins. Finally, we identified several auto-phosphorylated sites within SAPK kinase surface that might have a role in their interaction with PP2C and/or downstream partners.

## Materials and Methods

### Gene Identification and Bioinformatics Analyses

The sequences of ScSAPK8, ScSAPK9, and ScSAPK10 were identified using the Sugarcane Expressed Sequence Tag (SUCEST) database and the homologous sequences from *Sorghum bicolor* SbSAPK8: Sb01g007120, SbSAPK9: Sb08g019700 and SbSAPK10: Sb01g014720) and *Arabidopsis thaliana* (SnRK2.2: 824214, SnRK2.3: 836822, SnRK2.6: 829541) were used as reference (Vettore et al., 2003). The coding sequences of the three sugarcane SAPKs were isolated from the sugarcane leaf cDNA (cultivar SP80-3280) using specific primers (**Supplementary Table S1**).

For analysis of protein conservation, protein sequences from *A. thaliana*, *Zea mays* SAPK8 (NP_001149657.1), and *Saccharum* spp. were aligned using BioEdit and Clustal Omega (Hall, 1999; Sievers and Higgins, 2014). The sequence similarities, as well as the secondary structure elements, were further analyzed using the ESPript 3.0 program (Robert and Gouet, 2014). The analysis of protein domains was performed using PFAM and SMART databases (Schultz et al., 1998; Finn et al., 2016).

For phylogenetic analysis using MEGA7 software (Kumar et al., 2016), multiple sequence alignment was previously performed using MUSCLE server (Madeira et al., 2019). The phylogenetic tree was constructed using the Maximum Likelihood method, Jones-Taylor-Thornton model with invariant sites (Jones et al., 1992), 1000 times bootstrapping and gaps elimination.

### ScSAPKs Cloning and Recombinant Protein Expression in *Escherichia coli*

The full-length sequences of ScSAPK8/9/10 were cloned into pNIC28-Bsa4 using the ligase-independent cloning (LIC) method (Savitsky et al., 2010). For large scale protein expression, the constructs were transformed into *E. coli* strain BL21(DE3)-R3-pRARE2 (Savitsky et al., 2010) and grown at 37°C in 20 ml of LB medium with kanamycin (50 µg/ml). After overnight growth, the bacterial culture was inoculated into 1.5 L of Terrific Broth medium with kanamycin (50 µg/ ml), which was incubated at 37°C with shaking until an OD_600_ of 1.5. The culture was cooled to 18°C before the addition of 0.2 mM of IPTG (Isopropyl β-d-1-thiogalactopyranoside) for overnight expression. Cells were harvested by centrifugation at 7,500×*g* at 4°C and suspended in approximately 20 ml of 2× lysis buffer (100 mM HEPES pH 7.5; 1 M NaCl, 20 mM imidazole, 20% glycerol) with 1 µL per ml protease inhibitor cocktail. Suspended cells were placed on ice and sonicated for 9 min (5 s on; 10 s off; 30% amplitude). Polyethyleneimine (pH 7.5) was added to the lysate at 0.15% final concentration, and the lysate was clarified by centrifugation at 53,000 ×*g* for 45 min at 4°C. The supernatant was loaded onto an IMAC column (5 ml HisTrap FF Crude) and washed with Binding Buffer (50 mM HEPES pH 7.4, 500 mM NaCl, 5% glycerol, 10 mM imidazole pH 7.4, 0.5 mM tris(2-carboxyethyl)phosphine (TCEP)) and Wash Buffer (50 mM HEPES pH 7.4, 500 mM NaCl, 5% glycerol, 30 mM imidazole pH 7.4, 0.5 mM TCEP). The protein was eluted with 10 ml of Elution Buffer (50 mM HEPES pH 7.4, 500 mM NaCl, 5% glycerol, 300 mM imidazole pH 7.4, 0.5 mM TCEP) in 2-ml fractions. The eluted fractions were combined and incubated with TEV protease during overnight dialysis against GF Buffer (Binding Buffer without imidazole). TEV protease, as well as the cleaved 6xHis-tag, were removed using nickel-affinity chromatography resin. The protein was concentrated to 5 ml with a 30-kDa MWCO spin concentrator and loaded onto a size exclusion HiLoad 16/600 Superdex 200pg (GE) column equilibrated in GF buffer. Fractions of 1.8 ml were collected and verified for protein purity on a 12% SDS-PAGE gel. Purified fractions were combined, concentrated, and stored at −80°C.

### ScSAPK10 Crystallization, Data Collection and Structure Determination

For crystallization experiments, the truncated construct of ScSAPK10 corresponding to amino acids 12 to 320 (ScSAPK10_∆Nterm-∆ABA box) was cloned, and the recombinant protein produced as above. Before setting up crystallization trials, protein aliquots at 24 mg/ml were thawed and centrifuged at 15,000 rpm for 10 min at 4°C. Crystallization sitting drops were manually mounted using a 1:1 ratio of protein to reservoir solution. Crystals grew in 1.5M ammonium sulfate; 0.1M bis-tris pH 6.5 and 0.1M sodium chloride (reservoir solution) after 2 days at 20°C and were cryoprotected in reservoir solution supplemented with 30% glycerol before flash cooling in liquid nitrogen. Diffraction data were collected at the Advanced Photon Source (Chicago, USA) beamline 19ID. The X-ray diffraction data were integrated with XDS (Kabsch, 2010) and scaled using AIMLESS from the CCP4 software suite (Winn et al., 2011). The structure was solved by molecular replacement using Phenix (Adams et al., 2002) and the *A. thaliana* SnRK2.6 structure (PDB ID 3ZUT) as the initial model (Yunta et al., 2011). Refinement was performed using REFMAC5 (Murshudov et al., 2011). Coot (Emsley et al., 2010) was used for manual model building and local refinement. Structure validation was performed using MolProbity (Chen et al., 2010). Structure coordinates have been deposited in the Protein Data Bank (PDB ID 5WAX) (**Table 1**).

**Table 1:** Data collection and refinement statistics.

Protein	ScSAPK10
PDB ID	5WAX
**Data collection**	
X-ray source	APS 19-ID
Wavelength (Å)	0.979200
Space group	*C* 2 2 2_1_
Cell dimensions (Å) a, b, c.	75.4, 214.6, 93.8
Cell dimensions (°) α, β, γ.	90, 90, 90
Molecules/asymmetric unit	2
Resolution (Å)*	46.58–2.00 (2.05–2.0)
Unique reflections*	51563 (3763)
*R*_merge_ (%)*	8.3 (98.3)
I/σ (I)*	16.0 (1.6)
CC (1/2)*	0.999 (0.604)
Completeness (%)*	99.6 (99.9)
Redundancy*	5.5 (5.6)
	
**Refinement**	
Resolution (Å)*	46.58–2.00 (2.05–2.0)
*R*_cryst_/*R*_free_ (%)	19.83/23.7
No. atoms (protein/solvent)	4360/387
Mean B-factor (Å^2^)	29.9
Root mean square deviation (r.m.s.d.) bond lengths (Å), angles (°)	0.012, 1.49
	
**Ramachandran statistics (%)**	
Favored/allowed/outliers	96.3/3.7/0

*Values in parentheses represent the highest resolution shell.

### ScSAPK10 WT and ScSAPK10_∆Nterm-∆ABA Box Autophosphorylation Assay

The ScSAPK10 WT and ScSAPK10 ∆Nterm-∆ABA box were expressed as previously described. For the *in vitro* phosphorylation assay, the proteins were diluted in kinase buffer (25 mM HEPES pH 7.5; 12 mM MgCl_2_, 1 mM dithiothreitol [DTT]) to a final concentration of 30 μg and incubated with or without 5 mM ATP (Sigma—catalog A7699). After 1 h incubation at 37°C, 10 μl of aliquots were removed, and the reaction stopped by freezing in liquid nitrogen. Samples were analyzed by LC-MS.

### Site-Directed Mutagenesis and ScSAPK8 Expression for Phosphorylation Assays

The SnRK2 box and ABA box mutants were produced by site-directed mutagenesis with specific primers (**Supplementary Table S1**) using as template the full-length construct of ScSAPK8 in pNIC28-Bsa4. The mutated constructs were confirmed by sequencing and transformed in *E. coli* BL21(DE3)-R3 cells which express rare tRNAs (plasmid pACYC-LIC+) and the λ-phosphatase.

All proteins were expressed at the same time using the same protocol described previously. After bacterial culture lysis, the clarified supernatants were loaded in 4 ml of Ni^2+^-sepharose beads (GE Healthcare, Uppsala), washed with binding buffer (4 × 4 ml) and wash buffer (3 × 4 mL). The proteins were eluted with elution buffer (4 × 4 ml), and the imidazole was removed using Sephadex G-25 PD-10 Desalting Columns (GE Healthcare, Uppsala). Protein purity was analyzed by SDS-PAGE gel, and protein masses were confirmed by intact mass spectrometry.

### ScSAPK8 WT and Mutants Autophosphorylation Assay

Each protein (diluted in GF buffer to 20 μM final concentration) was incubated with 10 mM MgCl_2_ and 1 mM ATP (Sigma – catalog A7699) at 20°C in a final volume of 200 μl. After every time point (1 h, 5 h, and overnight), 20 μl of aliquots were removed, and the reaction stopped by the addition of 10 mM EDTA. Samples were analyzed by LC-MS. For these assays, the protein concentration was estimated by Bradford (Sigma-Aldrich) and SDS-PAGE analysis.

### Kinase Activity Assay

The enzymatic activity of ScSAPK8 WT and its SnRK2 box and ABA box mutants were measured using a TR-FRET based assay (Cisbio Kinease, catalog 62ST1PEB). Prior to testing the activity of wild-type and mutant proteins, assay conditions were optimized. For the enzymatic reaction, proteins were diluted in GF buffer supplemented with 10-mM MgCl_2_ to a 20-μM final concentration and incubated overnight at 20°C with or without 1 mM ATP. After 16 h, the activity of proteins pre-incubated with Mg^2+^/ATP and Mg^2+^ was tested using the peptide STK-1 at 1-μM final concentration. Final assay concentrations were: 50-nM kinase, 2-mM ATP, 10-mM MgCl_2_, and 1-mM DTT. The reaction was allowed to progress for 1 h at room temperature before the detection step was performed according to the manufacturer’s instructions. Fluorescence resonance energy transfer (FRET) signal was acquired using a ClarioStar fluorescence plate reader (BMG Labtech) (excitation/emission wavelengths of 330 and 620/650 nm, respectively). Results reported are from two independent experiments performed in triplicates.

The activity data are presented as mean ± SD. The difference of three or more groups to the wild type was compared using one-way analysis of variance (ANOVA) with Dunnett’s correction. Two-way ANOVA *post-hoc* Bonferroni was used to compare the mean of each protein with no pre-incubation with ATP and 16-h ATP incubation. In all cases, *P* < 0.05 was considered statistically significant. Statistical analyses were performed on Prism 8 (GraphPad).

### ScSAPK8 WT Phosphosite Identification

Purified ScSAPK8 WT and ∆ABA box mutant (20 μM final concentration) were diluted in GF buffer supplemented with 10 mM MgCl_2_ and incubated with 1 mM ATP (Sigma – catalog A7699) overnight at 20°C. The reaction was stopped by adding 10-mM EDTA (final concentration) before flash-freezing samples in liquid nitrogen. Protein intact mass was determined by LC-MS, and phosphosites were identified by LC-MS/MS. The sample was buffer-exchanged into 50-mM ammonium bicarbonate and treated with 25 µL of RapiGest SF (0.2%; Waters Corp. catalog 186001861) for 15 min at 80°C. DTT (100 mM stock prepared in 50 mM Ammonium Bicarbonate) was added to a final concentration of 4 mM, and the mixture was incubated for 30 min at 60°C. Iodoacetamide (IAA) (300 mM stock prepared in 50 mM Ammonium Bicarbonate) was added to the mixture at a final concentration of 12 mM. The mixture was protected from light and incubated for 30 min. Trypsin (catalog V511A; Promega, Fitchburg, WI, USA) prepared in 50 mM ammonium bicarbonate was added to the mixture (1:100 mass ratio of trypsin to protein) and incubated for 16 h at 37°C under agitation. To hydrolyze the RapiGest, trifluoroacetic acid (TFA) (catalog 53102; Pierce, Waltham, MA, USA) was added, and the mixture incubated for 90 min at 37°C. The reaction was centrifuged at 14,000 rpm for 30 min at 6°C, and the supernatant transferred to a fresh microcentrifuge tube (Axygen, Union City, CA, USA) for subsequent LC-MSMS analysis. Data have been deposited to the ProteomeXchange Consortium *via* the PRIDE (Perez-Riverol et al., 2019) partner repository with the data set identifier PXD014298.

### Mass Spectrometry Analysis

For intact mass analysis, samples were analyzed *via* reverse phase HPLC-ESI-MS in positive ion mode using an Acquity H-class HPLC system coupled to a XEVO G2 Xs Q-ToF mass spectrometer (both from Waters Corp.). A total of 0.5-μl sample (∼12.5 ng) in mobile phase Solvent A (0.1% formic acid [FA], prepared in water) was applied onto a C4 column (ACQUITY UPLC Protein BEH C_4_ 300 Å, 1.7 µm, 2.1 mm × 100 mm; Waters Corp.) kept at 45°C. Bound protein was eluted by a gradient of 10-90% Solvent B (0.1% FA in 100% acetonitrile [ACN]) over 4 min. Between each injection, the column was regenerated with 90% Solvent B (for 90 s) and re-equilibrated to 10% Solvent B (210 s). Flow rates were 0.5-µL/min for sample application and 0.4 ml/min (wash and elution). For internal calibration, the lockspray properties were: scan time of 0.5 s; and a mass window of 0.5 Da around Leu-enkephalin (556.2771 Da). The ToF-MS acquisition ranged from 100 to 2000 Da with a scan time of 1 s. The cone voltage on the ESI source was fixed at 40 V.

For phosphosite identification, samples were analyzed by reverse phase nanoLC-ESI-MSMS using an Acquity M-class HPLC system coupled to a XEVO G2 Xs Q-ToF (both from Waters Corp.). A total of 2-μl sample in mobile Solvent A was applied onto a Trap column (V/M, Symmetry C18, 100 Ǻ, 5 µm, 180 µm × 20 mm) connected to an HSS T3 C18 column (75 µm × 150 mm, 1.8 μm), kept at 45°C. LC was performed at a flow rate of 400 nL/min, and the elution of bound peptides was performed over a 47-min gradient as follows: 0-30.37 min from 7-40% Solvent B; 30.37-32.03 min from 40% to 85% Solvent B; 32.34-35.34 min at 85% Solvent B; 35.34-37 min from 85% to 7% Solvent B and 37-47 min at 7% B. The nano-ESI source was set with the following parameters: the capillary voltage was 2.5 kV, the sampling cone and the source offset was set at 30 V, the temperature source was 70°C, the gas flow and the purge gas were set at 50 and 150 L/h, and the nano gas flow was maintained at 0.5 bar. Data were acquired at 0.5 scan/s, over the mass range of 50 to 2000 m/z in positive and sensitive mode. The MS data-independent acquisition mode was used with a low energy collision switched off and a high collision energy ramp 15 to 45 eV in the second function for fragmentation. For mass accuracy, the Glu-Fibrinopeptide (785.84261 Da 2+) was used as lock mass at a concentration of 100 fM (in 40:60 ACN/H_2_O, 0.1% FA) infused at a flow rate of 0.5 µL/min *via* a lock spray interface and an auxiliary pump. Lock mass scans were acquired every 30 s at a rate of 0.5 scan/s. Lockmass was acquired but not applied on the fly.

### MS Data Analysis

MS raw data were analyzed using MassLynx v4.1 and processed by MaxEnt 1 (both from Waters Corp.) to deconvolute multi-charged combined ion spectra for intact mass analysis. Phosphoproteomic raw data were processed using the Protein Lynx Global Server (PLGS, Waters Corp.) against the sugarcane protein database (UniProt release 2017_12). Data processing was performed in two steps. First, PLGS extracted all acquired spectra using the following parameters: lock mass (charge 2 = 785.84261 Da/e) window set to 0.4 Da; low energy threshold fixed at 500 counts; elevated energy threshold at 50 counts; chromatographic peak width and MS ToF resolution were set to automatic. Then, a database search was performed with the following parameters: peptide and fragment tolerance were set to automatic; two fragments ion matches per peptide and five fragments ion matches per protein were fixed, as well as a minimum of one peptide match per protein; one missed cleavage was allowed; trypsin was set as the primary digestion; carbamidomethylation of cysteine was set as a fixed modification, oxidation of methionine, and phosphorylation of Ser/Thr/Tyr residues were set as a variable modification.

## Results

### ABA-Responsive SnRK2s in Sugarcane

Three ABA-responsive SnRK2s were identified within the sugarcane genome (*S*. *spontaneum*× *S*. *officinarum hybrid cultivar*) using homologous protein sequences from *A. thaliana* and *Sorghum bicolor*. Based on previous studies in monocots and eudicots, these were designated ScSAPK8, ScSAPK9, and ScSAPK10 (Boudsocq et al., 2004; Kobayashi et al., 2004; Fujii et al., 2009; Fujita et al., 2009; Li et al., 2010; Cai et al., 2014; Li et al., 2017).

**Figure 1:**
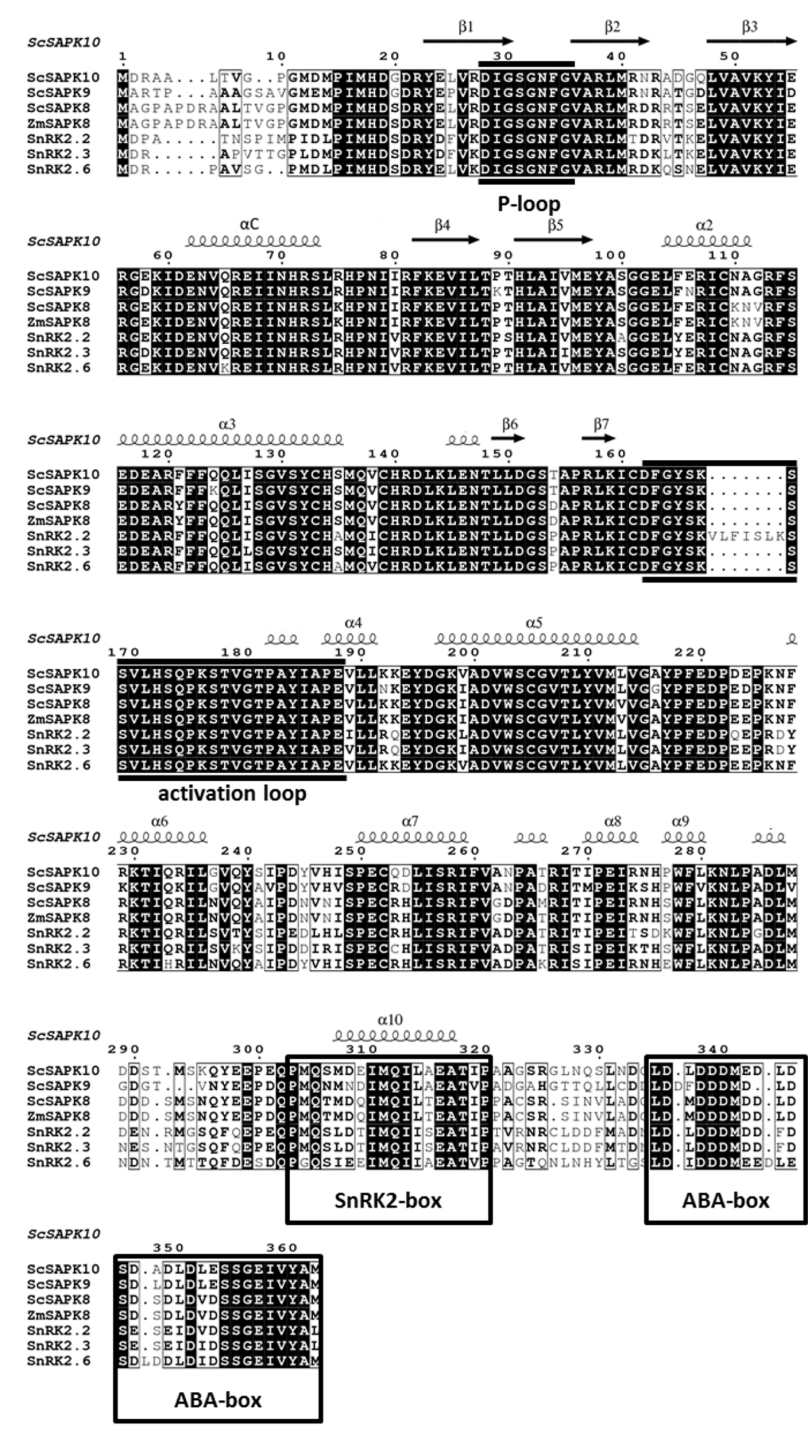
Multiple-sequence alignment of ABA-related SnRK2s shows high similarity and identity between Arabidopsis, maize and sugarcane sequences. Black boxes highlight identical residues, residues in black font have similar chemical properties. Sequences corresponding to the P-loop, activation loop and the regulatory domains SnRK2 box and ABA box are marked based on previous studies (Ng et al., 2011).

Phylogenetic analysis revealed that sugarcane SnRK2s present higher homology to *Z. mays* SAPK8 compared to *A*. thaliana proteins (**Supplementary Figure S1**). The ZmSAPK8 protein is homologous to Arabidopsis SnRK2.6 and preserves a fundamental role in the coordination of stomatal movements and drought stress response (Vilela et al., 2013; Wu et al., 2019). At the amino acid level, sugarcane SAPKs share high-sequence identity with ZmSAPK8 (≥78%) and *A. thaliana* proteins (≥71%) (**Figure 1**). Moreover, Arabidopsis ABA-responsive SnRK2s and their sugarcane counterparts display an identical modular architecture, in which the N-terminal kinase domain (KD; ∼260 amino acids) is followed by two highly conserved motifs—the SnRK2 box (16 amino acids) and ABA box (27 amino acids) (**Supplementary Figure S2**). The function of the N-terminal regions of each protein (about 20 residues) remains to be elucidated.

### Sugarcane and *A. thaliana* ABA-Responsive SnRK2s Share a Conserved Kinase Fold

To better understand the mechanism of sugarcane ABA-responsive SnRK2s, we pursued crystallization of all three ScSAPK proteins. Despite our best efforts, we could not obtain diffraction quality crystals of ScSAPK8 or ScSAPK9. To improve the diffraction quality of initial ScSAPK10 crystals, we used a truncated version of the protein (residues 12 to 320) in which residues at both N- and C-terminal regions were removed, including the ABA box. The protein structure was solved at 2.0 Å resolution by molecular replacement using the AtSnRK2.6 structure (PDB ID: 3ZUT) (Yunta et al., 2011) as a model (**Table 1**).

**Figure 2:**
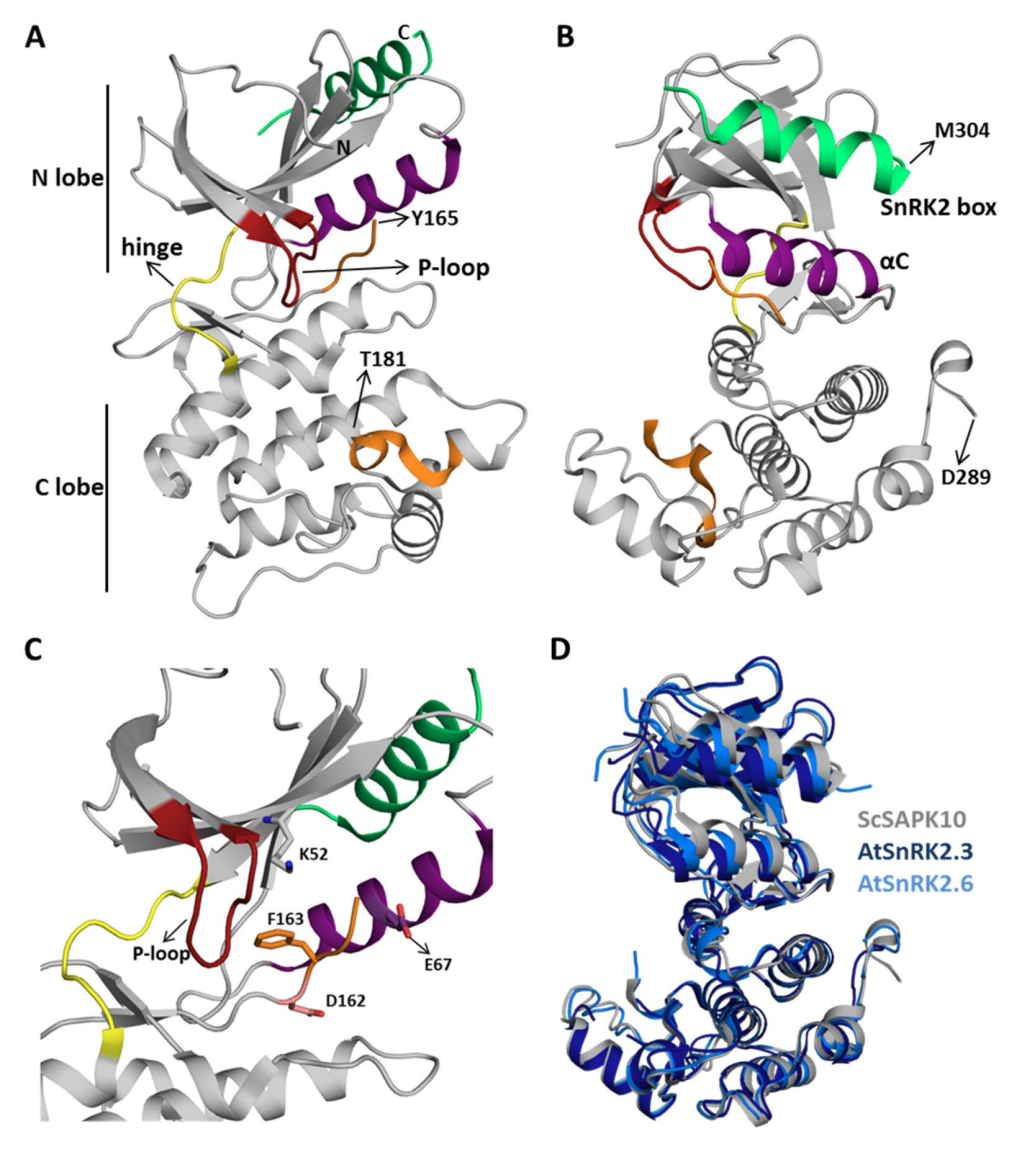
Sugarcane SAPK10 has a canonical kinase fold and a conserved SnRK2 regulatory domain. **(A** and **B)** Cartoon representation of the ScSAPK10 structure. Highlighted regions represent some of the key regions for kinase activity and/or regulation: ATP binding loop (red), αC (purple), activation loop (orange), and SnRK2 box (green). The hinge region that connects the N- and C-terminal lobes of the kinase domain is colored in yellow. Residues Y165-T181 and D289-M304 were not resolved in the electron density. **(C)** Cartoon representation of ScSAPK10 ATP-binding site. The ATP-binding loop (red), the activation loop residues D162 (pink) and F163 (orange), as well as the residues K52 (grey) and E67 (purple) related to phosphate transfer, are highlighted. **(D)** Structural alignment of ScSAPK10 (gray), Arabidopsis SnRK2.3 (PDB ID: 3UC3—dark blue), and SnRK2.6 (PDB ID: 3ZUT—light blue).

ScSAPK10 has a canonical kinase fold: a bilobal structure formed by a smaller N-terminal lobe and a larger C-terminal lobe connected by a short hinge region (**Figure 2A**). The protein N-terminal lobe is composed of five antiparallel β-strands, including the ATP-binding loop (P-loop) between β1 and β2, and the αC helix (Pearce et al., 2010). The C-terminal lobe contains the activation loop and several α-helices. Residues within the ScSAPK10 SnRK2 box (Met304-Pro320) are folded into an α-helix and packed against αC from the protein kinase domain (**Figure 2B**). No electron density was observed for residues in the activation loop (residues 165 to 181) or the region of the protein connecting the kinase domain and the SnRK2 box (residues 279 to 294), likely due to flexibility. These regions were omitted from the final model.

Superposition of ScSAPK10 onto the structures of AtSnRK2.3 and 2.6 (Ng et al., 2011; Yunta et al., 2011) confirmed our expectation that ABA-responsive SnRK2s from mono and eudicot plants are structurally similar—ScSAPK10 and AtSnRK2.3 root mean square deviation (r.m.s.d.): 2.32 Å; ScSAPK10 and AtSnRK2.6 r.m.s.d: 1.35 Å (**Figure 2D**). In the crystal, ScSAPK10 adopted an inactive conformation in which the side chain of Phe153 within the conserved kinase motif DFG points toward the ATP-binding site. In this inactive conformation, structurally conserved regions of the kinase domain important for phosphate transfer are kept apart. Moreover, the protein P-loop was found folded toward the kinase hinge region, an orientation that is likely to prevent binding of ATP (**Figure 2C**).

### SnRK2s Box Structure and Function Are Conserved Between ScSAPK and AtSnRKs

**Figure 3:**
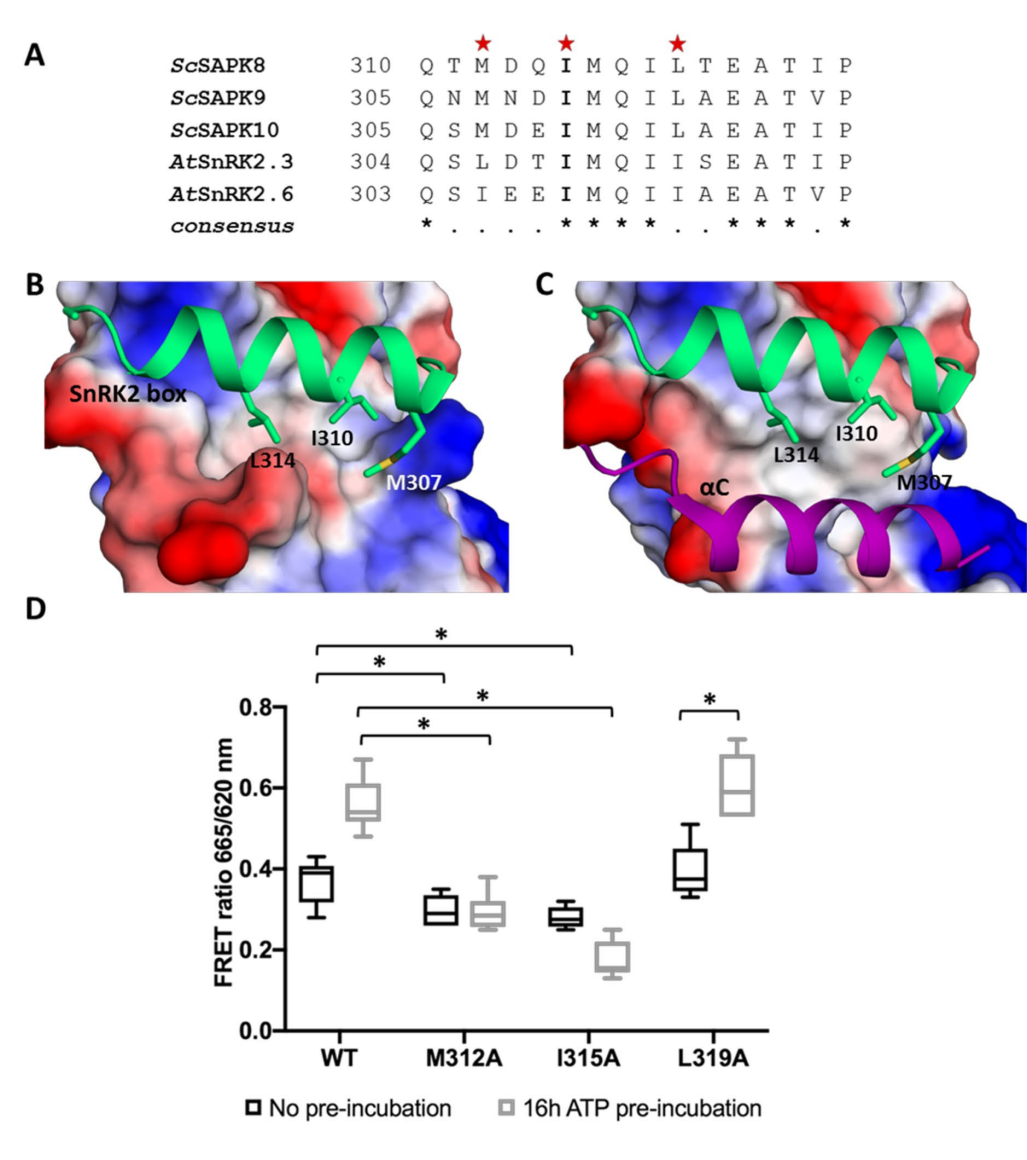
Key residues for SnRK2 box and αC helix interaction are conserved in ScSAPK10 and affect protein activity. **(A)** Alignment of SnRK2 box residues from sugarcane SAPKs, SnRK2.3, and SnRK2.6. Red stars represent the residues chosen for site-directed mutagenesis. The residue I315 is conserved in sugarcane and Arabidopsis SnRK2s while the residues M312 and Leu319 are conservatively substituted. **(B)** Cartoon representation of SnRK2 box (green) from ScSAPK*10_∆*nterm*-∆ABA-box* structure. ScSAPK10 SnRK2 box residues M307, I310, and L314 (homologous to ScSAPK8 M312, I315, and L319) are displayed as sticks and make close contact with the αC helix surface. The electrostatic potential analysis shows the negative potential (in red) of the αC surface. The positive potential is represented in blue. **(C)** Cartoon representation of SnRK2 box (green) and the αC helix (purple) from ScSAPK*10_∆*nterm*-∆ABA-box* structure. The electrostatic potential of αC surface was hidden to show the helix position. **(D)** Box plot of the enzymatic activity of ScSAPK8 WT and the mutants M312A, I315A and L319A after ATP incubation. The data show the quantity of phosphorylated peptide produced, measured by the ratio of fluorescence intensity at 665 nm (streptavidin-XL665 emission excited by phospho-specific Eu-cryptate conjugated antibody) and 620 nm (Eu-cryptate emission). In both assay conditions, the observed activity for ScSAPK8 WT was significantly higher than the mutants M312A (*p = 0.0416 for no ATP pre-incubation and *p < 0.0001 for 16 h ATP pre-incubation) and I315A (*p = 0.0114 for no ATP pre-incubation and *p < 0.0001 for 16 h ATP pre-incubation). The L319A activity was similar to WT in both conditions but significantly increased with 16 h of ATP pre-incubation (*p < 0.0001).

As seen for other SnRK2 family members, ScSAPK10 SnRK2 box is packed against the αC helix, within the protein kinase domain (**Figure 2**). Contacts between αC and the SnRK2 box are facilitated by conserved amino acids bearing aliphatic side chains (**Figures 3A–C**). Previous studies have shown that single-point mutations disturbing hydrophobic interactions between αC and SnRK2 box decreased kinase activity of AtSnRK2s (Ng et al., 2011). Considering the high levels of conservation between ScSAPKs and their *A. thaliana* counterparts, we decided to investigate if a similar mechanism could regulate the activity of the sugarcane proteins.

To measure the activity of recombinantly expressed ScSAPKs, we employed a commercially available enzymatic assay (KinEASE, Cisbio) and a generic peptide substrate (STK1 from the same vendor). As can be seen in **Supplementary Figure S3**, phosphorylation of the peptide substrate increased with increasing concentrations of ScSAPK8. Pre-incubation of the enzyme with ATP also increased ScSAPK8 kinase activity. We, thus, employed this assay to study the impact of perturbing the interaction between the protein SnRK2 box and αC helix on the activity of ScSAPK8.

We used site-directed mutagenesis to substitute conserved SnRK2 box residues (Met312, Ile315, or Leu319) with an alanine. All proteins were expressed and purified side by side and had their purity and concentration evaluated spectroscopically and by SDS-PAGE (**Supplementary Figure S4**). We first performed enzymatic assays to compare the overall activity of wild-type and mutant ScSAPK8. Our results showed that the activities of ScSAPK8 mutants M312A and I315A were lower than that of the wild-type enzyme (one-way ANOVA post hoc Dunnett’s, n = 6, *p* = 0.0416 comparing WT to M312A and *p* = 0.0114 for WT and I315A; ANOVA *p* = 0.0011), whereas the activity of the L319A mutant was comparable to that of the wild-type protein (*p* = 0.7021) (**Figure 3D**).

AtSnRK2s are known to be activated by autophosphorylation (Fujii et al., 2009; Ng et al., 2011). We then investigated the impact of pre-incubating wild-type and mutant ScSAPK8s with ATP (16 h at 25°C) before assaying their activity. Pre-incubation with ATP increased the activity of the wild-type protein and that of the L319A mutant (two-way ANOVA post hoc Bonferroni’s, *p* < 0.0001, n = 6), whereas the activity of mutant M312A remained unaltered (*p* > 0.9999). Surprisingly, the overall activity of I315A was reduced (*p* = 0.0034) (**Figure 3D**).

To verify if the reduced activity observed for ScSAPK8 mutants M312A and I315A resulted from the inability of these proteins to self-activate, we used LC-MS to obtain the intact masses of wild-type and mutant proteins. The total number of phosphosites observed after 16-h incubation with ATP remained the same for wild-type and mutant proteins M312A and I315A (**Supplementary Figures S5–S7**). Thus, the reduced activities observed for these two mutant proteins were not due to a defect in their auto-phosphorylation abilities. Surprisingly, mutant L319A displayed three additional phosphosites compared with the wild-type and the other two mutants investigated (**Table 2**; **Supplementary Figures S5** and **S8**).

**Table 2:** Intact mass analysis of ScSAPK8 proteins after overnight incubation with Mg^2+^/ATP.

Construct	Total number of phosphorylations
**ScSAPK8-WT**	4
**ScSAPK8-M312A**	4
**ScSAPK8-I315A**	4
**ScSAPK8-L319A**	7
**ScSAPK8 ABA box group 1**	7
**ScSAPK8 ABA box group 2**	7
**ScSAPK8 ABA box group 3**	7
**ScSAPK8 ABA box group 4**	7
**ScSAPK8 ∆ABA box**	1

Taken together, our results indicate that mutations on Met312 and Ile315 designed to perturb the interaction between the αC helix and SnRK2s box in ScSAPK8 reduced overall protein activity.

### Deletion of SAPK8 ABA Box Does Not Directly Affect Its Activity

**Figure 4:**
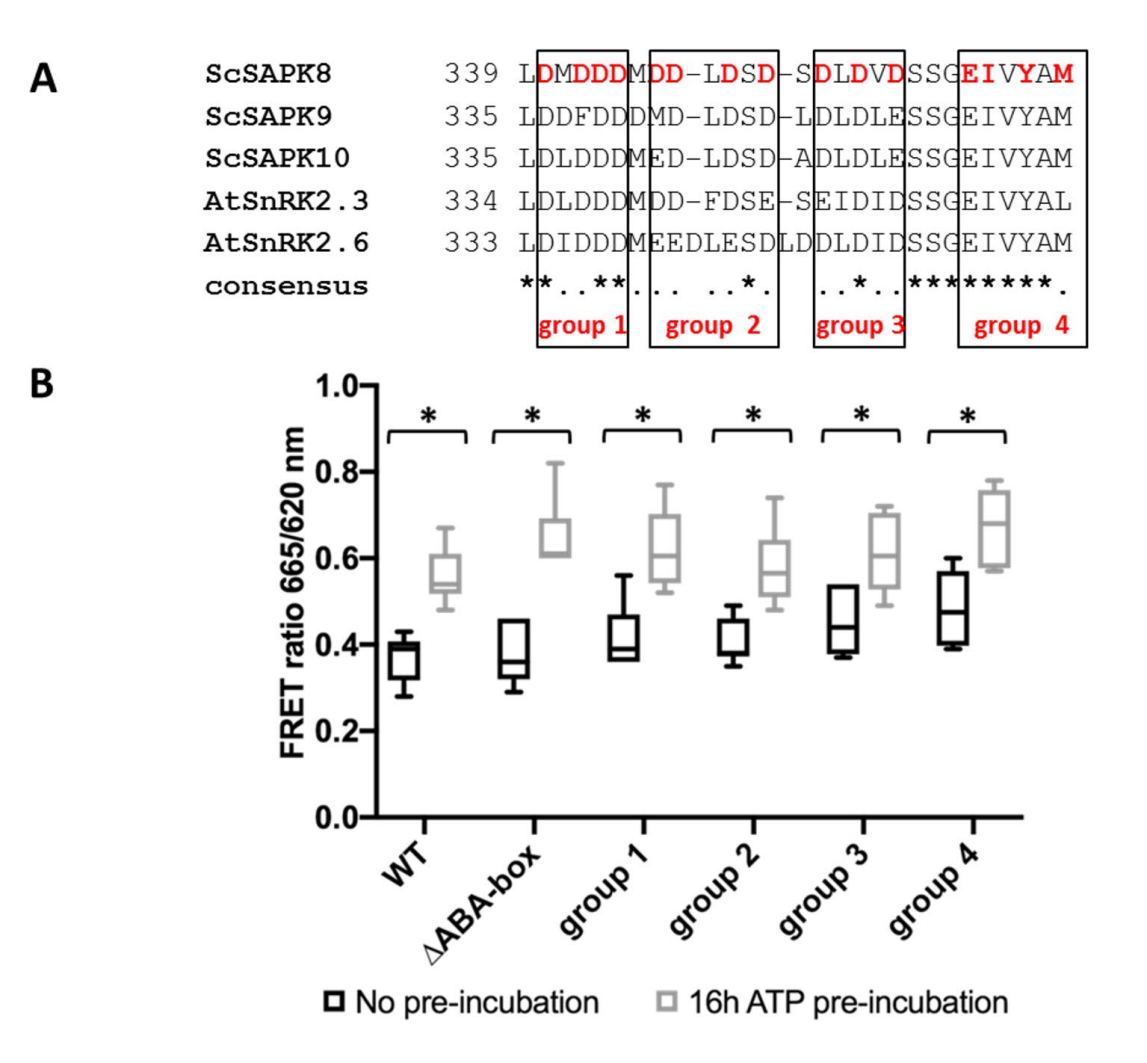
ScSAPK8 ABA box mutations do not affect protein activity. **(A)** Alignment of ABA box residues from sugarcane SAPKs and SnRK2.6. Mutations performed in ScSAPK8 ABA box are displayed in red and were distributed in four different groups, named group 1 to group 4. In the mutants from group 1 to group 3 all aspartic acid residues (D, in red) were replaced by alanine residues. In group 4, the residues of glutamic acid, isoleucine, tyrosine and methionine (respectively, E, I, Y and M, in red) were mutated to alanine **(B)** Box plot of ScSAPK8 WT and ABA box mutant enzymatic activity after ATP incubation. The data show the quantity of phosphorylated peptide produced, measured by the ratio of fluorescence intensity at 665 nm (streptavidin-XL665 emission excited by phospho-specific Eu-cryptate conjugated antibody) and 620 nm (Eu-cryptate emission). The analysis shows no statistically significant difference between the activity of WT and all the mutants tested. All the proteins presented significantly increased activity after 16 h of ATP pre-incubation compared to the condition with no pre-incubation (*p* < 0.0001).

In addition to the SnRK2 box, another conserved C-terminal region is involved in SnRK2 regulation in eudicot plants: the ABA box (Belin et al., 2006; Boudsocq et al., 2007; Soon et al., 2012). This region mediates the interaction between SnRK2s and PP2C phosphatases, leading to kinase inactivation *via* dephosphorylation of an essential activation loop serine residue and preventing substrate access to the kinase catalytic site (Belin et al., 2006; Soon et al., 2012). Here, we investigated ScSAPK8 ABA box contribution to kinase activity in the absence of a PP2C phosphatase. For that, we used the enzymatic assay described above to assess the activity of several ScSAPK8 C-terminal mutants designed to either completely remove the protein ABA box or to disrupt the region’s acidic character *via* replacement of conserved acidic residues with alanine residues (**Figure 4A**).

All ScSAPK8 mutants displayed similar overall activity to the wild-type protein. Likewise, pre-treatment with ATP (16 h at 25°C) significantly increased enzyme activity for all proteins tested (two- way ANOVA post hoc Bonferroni’s, *p* < 0.0001, n = 6) (**Figure 4B**). We also used LC-MS to assess the impact of ScSAPK8 ABA box on protein auto-phosphorylation. Interestingly, more phosphosites could be detected for ScSAPK8 point mutants than for the wild-type protein (seven versus four phosphosites, respectively) (**Table 2**; **Supplementary Figures S5** and **S10–S13**). On the other hand, a single phosphorylation was detected in the truncated version of ScSAPK8 completely lacking the ABA box (**Table 2** and **Supplementary Figure S9**). Similar results were observed for the ScSAPK10 ΔABA box protein (**Supplementary Figure S14**).

Previously, phosphorylation of a serine residue (Ser175) within AtSnRK2.6 activation loop was described as important for protein activity (Ng et al., 2011). We then used LC-MS/MS to identify phosphosites within wild-type and ΔABA box ScSAPK8 proteins (**Table 3**). We identified five phosphorylation sites within the wild-type protein, including serine 182 located in the protein activation loop and structurally equivalent to AtSnRK2.6 Ser175. Other phosphorylation sites are located to the protein P-loop (Ser36), C-lobe (Ser120), activation loop (Thr186), and SnRK2 box (Thr320).

**Table 3:** ScSAPK8 phosphopeptides identification by mass spectrometry.

Kinase	Phosphorylated residue	Residue location	Total number of phosphorylations
**ScSAPK8-WT**	S36	P-loop	5
	S120	C-lobe	
	S182	activation loop	
	T186	activation loop	
	T320	SnRK2 box	
**ScSAPK8 ΔABA box**	S36	P-loop	2
	S182	activation loop	

Likewise, we identified Ser182 as a phosphosite within the ΔABA box ScSAPK8 mutant, indicating that this key activation loop residue is autophosphorylated in the mutant protein. Surprisingly, a second phosphosite was also identified within the mutated protein—Ser36.

Taken together, the data above suggest that ScSAPK8 ABA box is important for protein overall phosphorylation pattern, but, by itself, this conserved motif does not seem to regulate enzyme activity.

### Structure of SAPK10 Suggests a Conserved Interaction Mechanism With PP2C-Type Phosphatases

**Figure 5:**
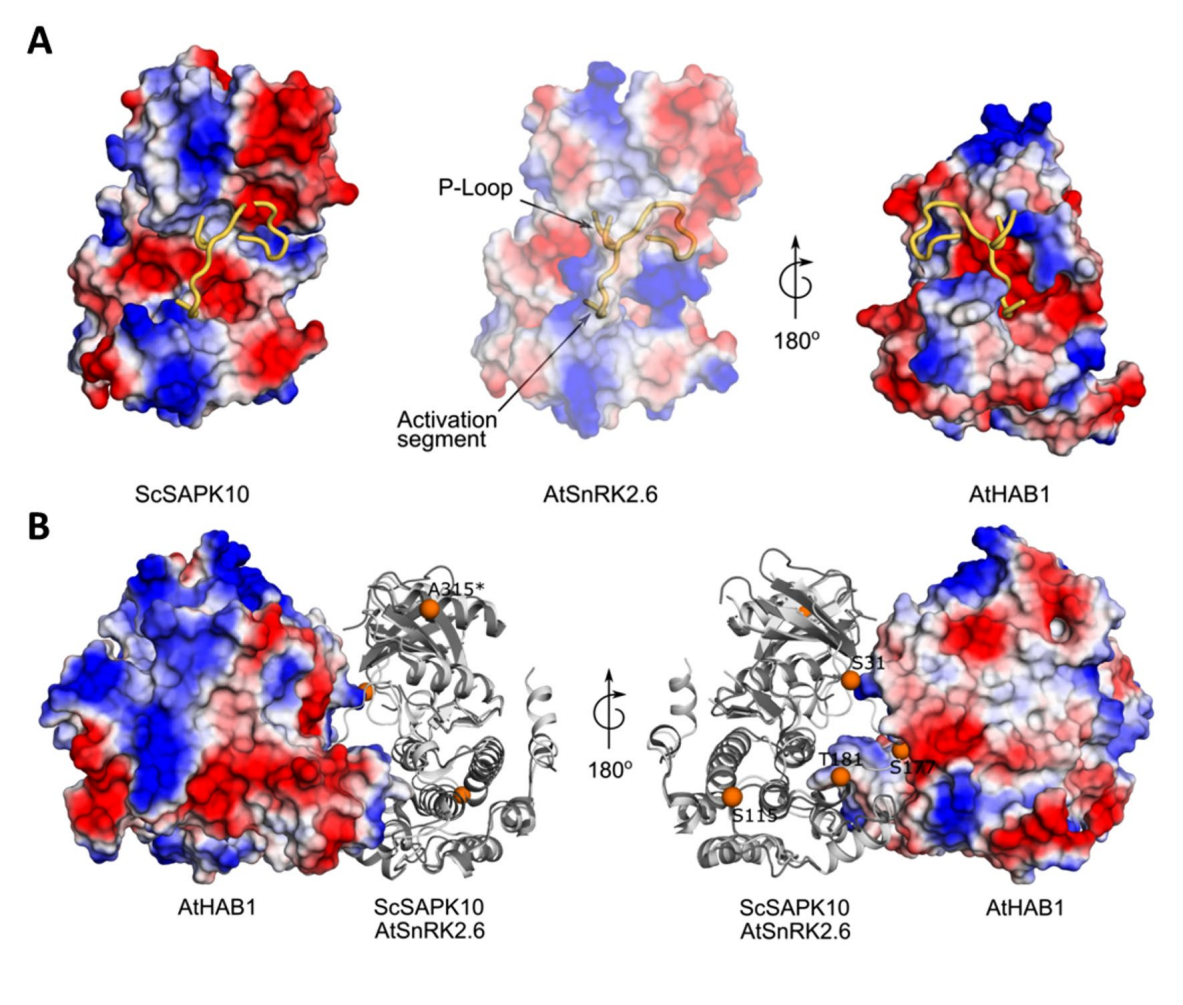
Structure of ScSAPK10 suggests a conserved interaction surface with PP2C-type phosphatases. **(A)** Cartoon representation of ScSAPK10, AtSnRK2.6, and AtHAB1 protein surfaces. The electrostatic potential analysis shows the positive potential (in blue) of the protein surfaces around the activation segment and P-loop. **(B)** Cartoon representation of ScSAPK10 (dark gray) aligned with AtSnRK2.6 (light gray) and AtHAB1 (represented as electrostatic surface). The orange spheres represent, in the ScSAPK10 structure, the homologous phosphosites identified to ScSAPK8 by mass spectrometry. The ScSAPK10 residues S31, S115, S177, T181, and A315 correspond to S36, S120, S182, T186, and T320 in ScSAPK8 sequence, respectively.

Structural studies revealed that AtSnRK2.6 and PP2C-type phosphatases display complementary electrostatic surfaces at the complex interface (Soon et al., 2012). Superposition of our ScSAPK10 structure to that of AtRK2.6 bound to a PP2C-type phosphatase (AtHAB1), revealed that both kinases display similar electrostatic surfaces within the SnRK2.6 region known to interact with PP2C-type phosphatases (**Figure 5A**).

We also mapped the identified phosphosites in wild-type ScSAPK8 onto our ScSAPK10 crystal structure. Three (Ser36, Ser182, and Thr186) of the five identified phosphosites in wild-type ScSAPK8 have structural equivalents in the Arabidopsis protein that are within the kinase:phosphatase complex interface (**Figure 5B**) (Soon et al., 2012).

These analyses suggest that the overall mechanism regulating the interaction of ABA-responsive kinases and PP2C-type phosphatases might be conserved between Arabidopsis and sugarcane proteins.

## Discussion

ABA is a key hormone in both mono- and eudicotyledon plants. In eudicots, members of the SnRK2 family of protein kinases play a central role in ABA signaling and act as positive regulators of this stress hormone (Fujii and Zhu, 2009; Fujii et al., 2009; Fujita et al., 2009; Nakashima et al., 2009). It is expected that the signaling pathway relaying ABA stimuli is also conserved in monocot plants. Supporting this hypothesis, recent studies have shown that ABA strongly activates expression of SnRK2 counterparts in sugarcane (Li et al., 2017). Here we confirmed that three functional SnRK2 proteins—ScSAPK8, ScSAPK9, and ScSAPK10—are encoded by the sugarcane genome, further suggesting that the ABA-response pathway is conserved in both mono- and eudicotyledons.

Our analyses of the ScSAPK structure and biochemistry strongly suggest that ABA-responsive kinases in sugarcane are functionally equivalent to their counterparts in Arabidopsis. The structure of ScSAPK10 revealed that the C-terminal SnRK2 box folds into an α-helix and interacts with the structurally conserved αC from the protein kinase domain. A similar interaction has been reported for AtSnRK2 proteins and is thought important for kinase activity, akin to the activation mechanism of human cyclin-dependent kinases (Jeffrey et al., 1995; Ng et al., 2011). Nevertheless, the ScSAPK structure determined here and previously determined SnRK2 structures have captured the protein in its inactive kinase state, despite the observed interaction between αC and SnRK2 box. Obtaining the structure of an SnRK2 family member in a fully active conformation would shed light on how SnRK2 box contributes to protein activity.

Biochemical assays have shown that disrupting key hydrophobic contacts between αC and SnRK2 box *via* point mutations in AtSnRK2 can nearly abolish protein activity (Ng et al., 2011). Likewise, ScSAPK8 kinase activity was reduced, albeit not dramatically, by replacement of Met312 or Ile315 within the protein SnRK2 box with alanine residues. In Arabidopsis SnRK2.6, two mutations within the protein SnRK box were particularly important for nearly abolishing protein activity: I308R and I312A. Here, the equivalent ScSAPK8 mutants were I315A and L319A, respectively. In ScSAPK8, the L319A mutant displayed similar activity levels to the wild-type enzyme, whereas the I315A mutant displayed decreased activity. Discrepancies between our results and those obtained previously for AtSnRK2s might be due to differences in enzyme preparation and assay conditions. Further, we cannot discard the possibility that the introduced mutations did not completely disrupt the SnRK2 box αC interaction in ScSAPK8. Nevertheless, our results here corroborate previous findings that showed that perturbing the interaction between SnRK2 box and the kinase domain impact SnRK2 protein activity.

A second region important for regulating SnRK2 activity is the ABA box. This region is a stretch of mostly acidic residues that mediate the direct interaction between SnRK2 proteins and basic patches on the surface of PP2C-type phosphatases (Soon et al., 2012). This interaction prevents the phosphorylation activity of SnRK2s. *In vitro*, total deletion of AtSnRK2.6 ABA box did not affect protein activity. However, ectopic expression of this truncated protein in Arabidopsis *snrk2.6* mutants could not restore stomatal closure response. These same studies revealed that phosphorylation of sites within the kinase domain was important for promoting wild-type response to ABA (Belin et al., 2006; Yoshida et al., 2006).

Our data indicated that deleting ABA box from ScSAPK8 reduced the overall number of autophosphorylation sites within this protein—from five in the wild-type protein to two in the ΔABA box mutant. Nevertheless, deletion of ScSAPK8 ABA box did not alter kinase activity on a generic peptide substrate, suggesting that autophosphorylation of residues located in the P-loop (Ser36) and activation loop (Ser182) might be sufficient for full kinase activity *in vitro*. However, the lack of activity of the ΔABA box SnRK2 mutant in Arabidopsis might indicate a role for the additional phosphorylation events in kinase activity *in vivo*.

Interestingly, our analysis of wild-type ScSAPK8 and its ΔABA box mutant identified two phosphosites (**Table 3**), in contrast to our results of protein intact mass, which revealed a single phospho-state for these proteins (**Table 2**). The intact mass analysis captures the overall phospho-state of a protein mixture while the peptide analysis allows the precise identification of phosphosite position. Combined, these results might indicate that Ser36 and Ser182 phosphorylation do not co-exist in the same protein molecule. In this scenario, the intact mass analysis showed a single phospho-state corresponding, in fact, to a mixture of either one of the two different phosphosites. The underlying mechanism behind these observations is not clear at this moment and will require further investigation.

The activation loop is a structurally conserved feature of protein kinases, and phosphorylation of key residues within this region stabilizes the protein in an active conformation (Nolen et al., 2004). In Arabidopsis SnRK2s, phosphorylation of a serine residue (Ser175 in SnRK2.6) within the protein activation loop is essential for kinase activation (Belin et al., 2006; Boudsocq et al., 2007; Vlad et al., 2009; Ng et al., 2011). We identified the equivalent residue in ScSAPK8 as phosphorylated after incubation with Mg^2+^/ATP, further suggesting that SnRK2 family members from both monocots and eudicots display similar regulatory mechanisms.

## Conclusion

Here, we determined the crystallographic structure and performed the biochemical characterization of ABA-related SnRK2 proteins from the crop plant sugarcane. Our analyses suggest a role for ScSAPK ABA box in protein autophosphorylation pattern but not in overall enzyme activity. We also found that perturbing the SnRK2 box:αC interaction reduced the overall activity of ScSAPK8 on a generic peptide substrate. Future studies are required to evaluate the role of these two conserved regions, as well as that of the multiple phosphosites identified here, in kinase activation and activity *in planta*. 

## Data Availability

The coordinates and structure factors for ScSAPK10 crystal structure reported here have been deposited in the Protein Data Bank with accession code 5WAX. Proteomics data with identified phosphopeptides are available *via* ProteomeXchange with identifier PXD014298.

## Ethics Statement

The study was performed in accordance with the Declaration of Helsinki. Ethical approval was obtained from the Noguchi Memorial Institute for Medical Research Institutional Review Board, University of Ghana, Accra (NMIMR-IRB-CPN 006/16-17 revd. 2018), and the University of Cape Town's Human Research Ethics Committee, reference numbers 104/2018 and 621/2017. Written informed consent was obtained from all participants, if they were 18 years or older, or from the parents/guardians with verbal assent from children.

## Author Contributions

GR participated in all parts of the project. DS performed all mass spec analysis. LH collected diffraction data. CC helped with protein expression and purification, statistical and phylogenetic analyses. OG coordinated the design of expression constructs. RC coordinated crystal structure determination and refinement. KM, JE, MM, and RC coordinated the project. GR and RMC wrote the manuscript. All authors revised the manuscript.

## Funding

This work was supported by the Brazilian agencies FAPESP (Fundação de Amparo à Pesquisa do Estado de São Paulo) (2013/50724-5, 2013/155765 and 2014/50897) and CNPq (Conselho Nacional de Desenvolvimento Científico e Tecnológico) (465651/2014-3). The SGC is a registered charity (number 1097737) that receives funds from AbbVie, Bayer Pharma AG, Boehringer Ingelheim, Canada Foundation for Innovation, Eshelman Institute for Innovation, Genome Canada, Innovative Medicines Initiative (EU/EFPIA) [ULTRA-DD grant no. 115766], Janssen, Merck KGaA Darmstadt Germany, MSD, Novartis Pharma AG, Ontario Ministry of Economic Development and Innovation, Pfizer, Takeda, and Wellcome [106169/ZZ14/Z]. Germanna Righetto received fellowships from CAPES (Coordenação de Aperfeiçomento de Pessoal de Nível Superior) (33003017024P2) and CNPq (141368/2018-7). CC received a CAPES INCT fellowship (88887.158494/2017-00).

## Conflict of Interest Statement

The authors declare that the research was conducted in the absence of any commercial or financial relationships that could be construed as a potential conflict of interest.
