# Supplementary material for: The C-Terminal Domains SnRK2 Box and ABA Box Have a Role in Sugarcane SnRK2s Auto-Activation and Activity
Source: Front Plant Sci. 2019 Sep 17;10:1105. doi: 10.3389/fpls.2019.01105 (PMC6759772; doi:10.3389/fpls.2019.01105)
Supplement: Supplementary file 1 [file DataSheet_1.pdf]

The C-terminal domains SnRK2-box and ABA-box have a role in sugarcane SnRK2s auto-activation and activity

Germanna Lima Righetto [1], Dev Sriranganadane [2,3], Levon Halabelian [4], Carla G. Chiodi [2,3], Jonathan M. Elkins [3,5], Katlin B. Massirer [2,3], Opher Gileadi [5], Marcelo Menossi [1], Rafael M. Couñago [2, 3,*]

1 Functional Genome Laboratory, Department of Genetics, Evolution, and Bioagents, Institute of Biology, State University of Campinas, Campinas, SP, Brazil.

2 Centro de Química Medicinal (CQMED), Centro de Biologia Molecular e Engenharia Genética (CBMEG), Universidade Estadual de Campinas (UNICAMP), Campinas, SP, 13083-875, Brazil.

3 Structural Genomics Consortium, Departamento de Genética e Evolução, Instituto de Biologia, UNICAMP, Campinas, SP, 13083-886, Brazil

4 Structural Genomics Consortium, MaRS Centre, South Tower, 101 College St., Suite 700, Toronto, ON, M5G 1L7, Canada.

5 Structural Genomics Consortium, Nuffield Department of Medicine, University of Oxford, Oxford OX3 7DQ, UK.

* Corresponding author: Rafael M. Couñago (rafael.counago@unicamp.br)

SUPPLEMENTARY MATERIAL

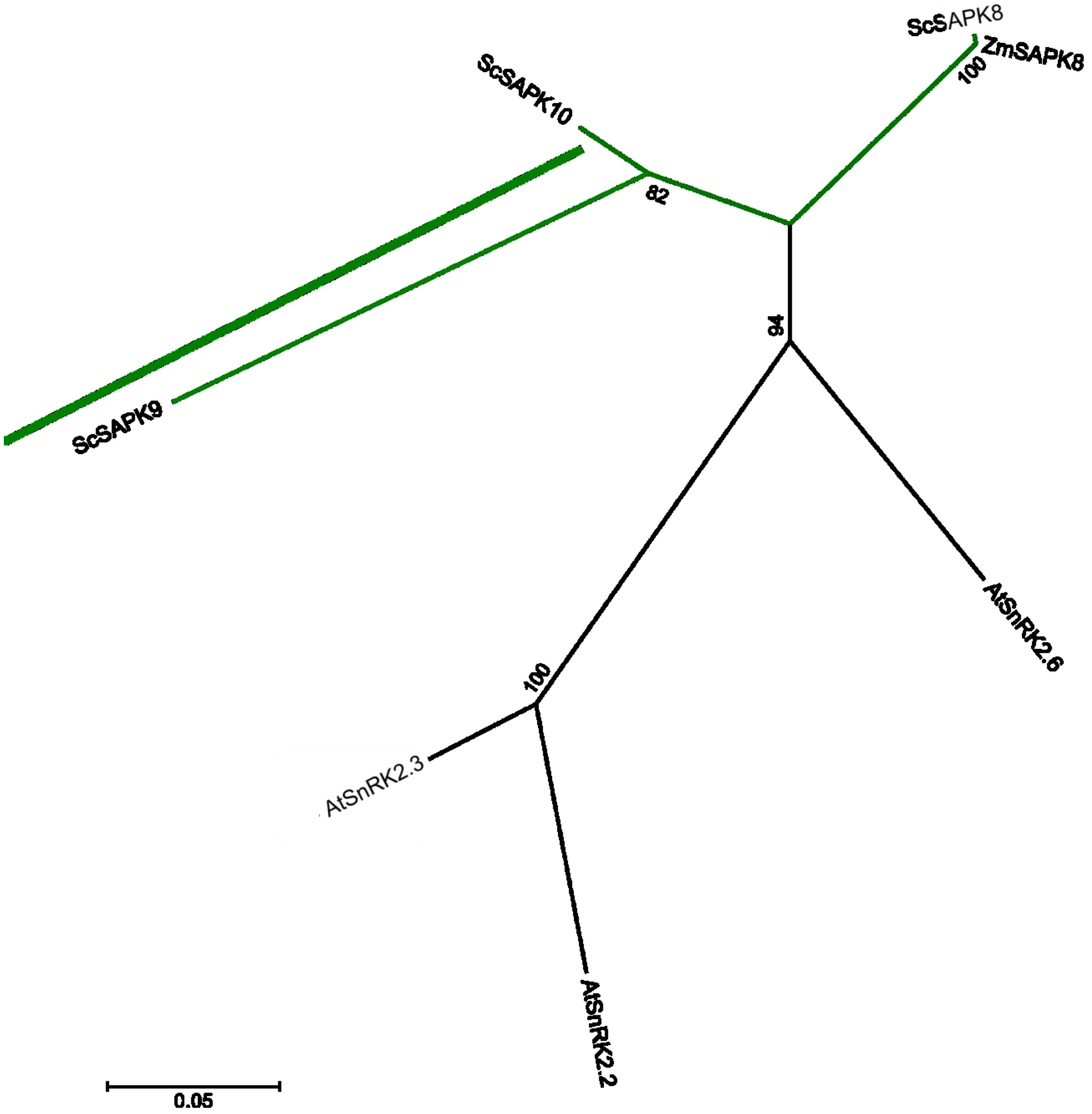

Supplementary Figure S1: Unrooted phylogenetic tree of sugarcane, maize and Arabidopsis SnRK2s inferred by Maximum Likelihood. The tree is drawn to scale, with branch lengths representing the number of substitutions per site. Bootstrapping analysis was performed 1000 times, and the values (in %) are shown at each node. Values above 80% indicate branches are well supported by our phylogenetic reconstruction. Branches corresponding to the conserved monocotyledons SnRK2 proteins are colored in green.

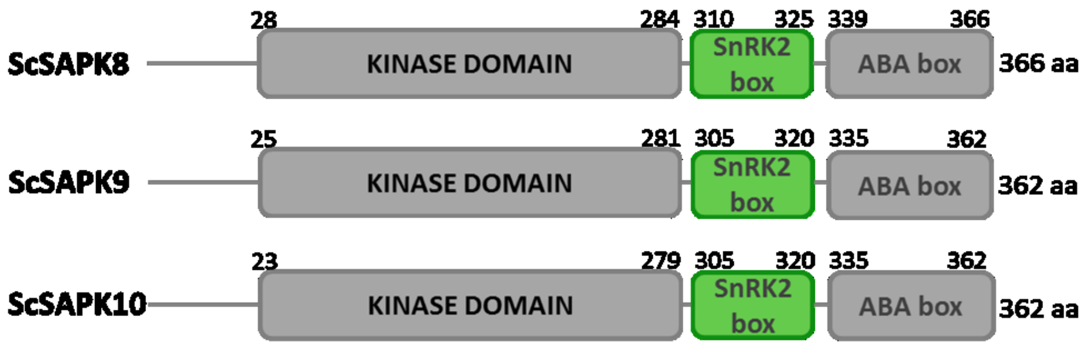

Supplementary Figure S2: Schematic representation of the full-length ScSAPK8, ScSAPK9, and ScSAPK10. Each protein has an N-terminal kinase domain and a C-terminal region containing the regulatory domains SnRK2-box and ABA-box. Numbers represent the amino acid positions of each protein domain.

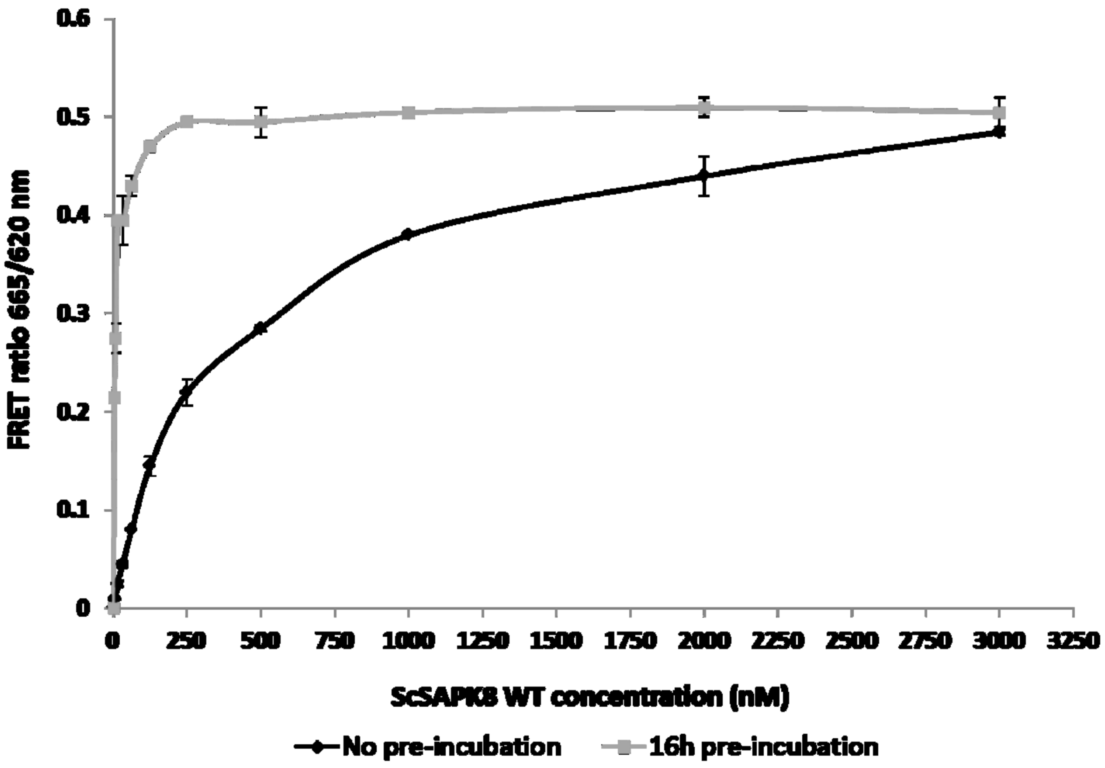

Supplementary Figure S3: Enzymatic activity of ScSAPK8 WT under variable enzyme concentration and after ATP pre-incubation. The data show the quantity of phosphorylated peptide produced after 1 hour, measured by the ratio of fluorescence intensity at 665 nm (streptavidin-XL665 emission excited by phospho-specific Eu-cryptate conjugated antibody) and 620 nm (Eu-cryptate emission). The ScSAPK8 activity was higher with ATP pre-incubation and also increased in an enzyme concentration-dependent manner.

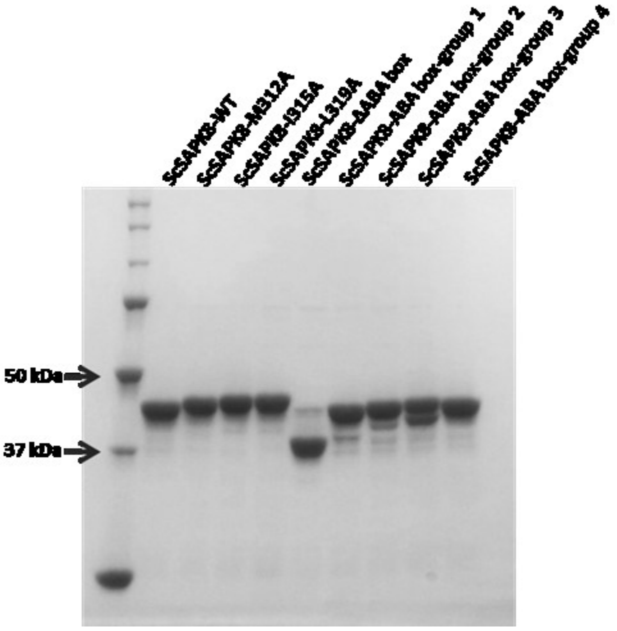

Supplementary Figure S4: Coomassie-stained SDS-PAGE of ScSAPK8 WT and mutants. The image represents all the proteins after affinity purification and dilution to 20 µM final concentration. The protein concentration was estimated by the Bradford method (Sigma-Aldrich) before gel loading.

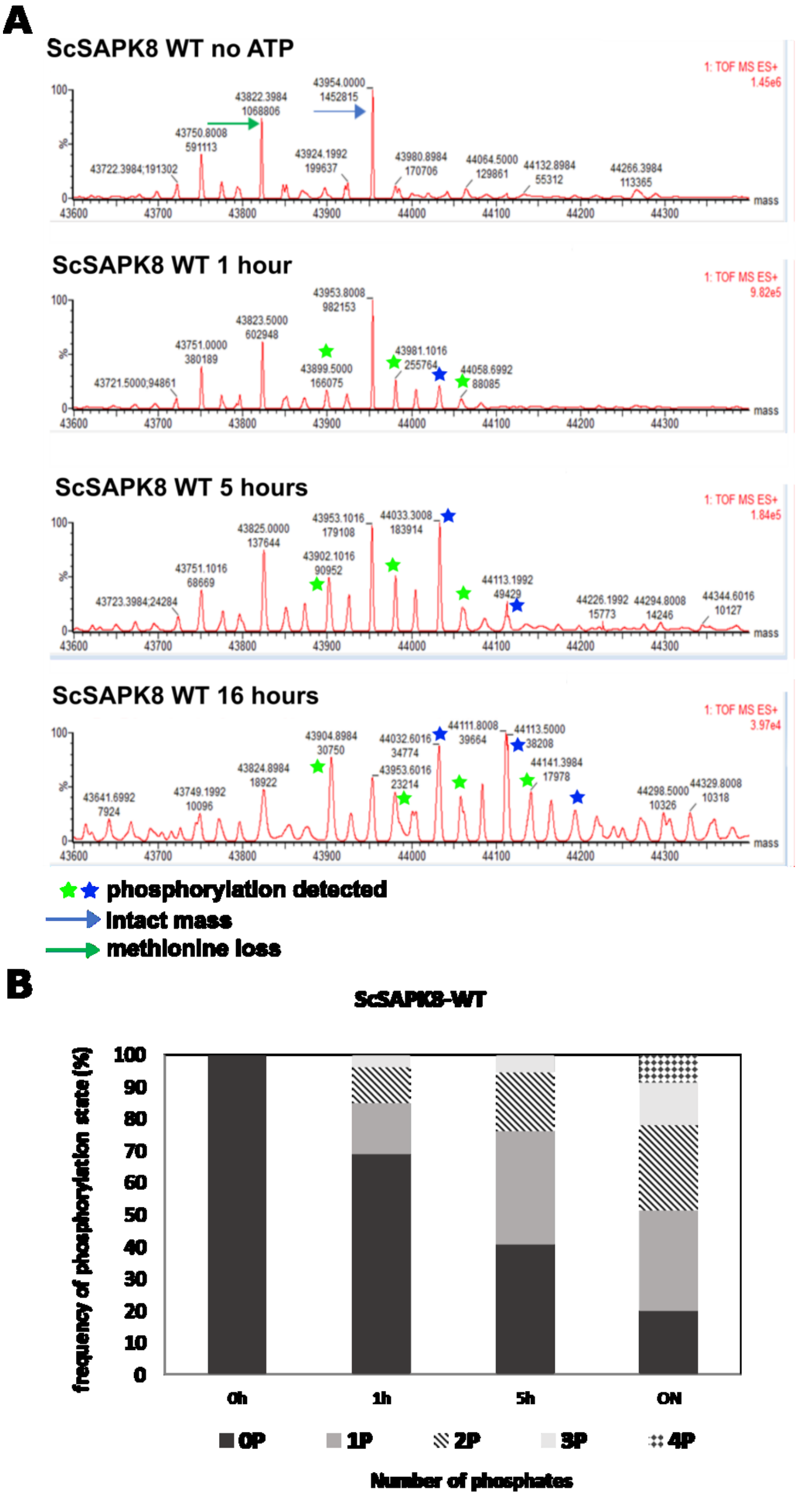

Supplementary Figure S5: ScSAPK8 WT autophosphorylation. A: Deconvoluted mass spectrum at time 0, 1 hour, 5 hours and overnight, generated by MAX ENT1 software (Waters). The blue arrow represents the intact kinase mass, and the blue stars represent the detected masses with one or more phosphorylations. The green arrows indicate the exact kinase mass with the loss of methionine that could occur during protein expression. The green stars represent masses of this kinase form with one or more phosphorylations. **B:** Graphical representation of protein autophosphorylation over time. The percentage values were calculated using the ion counts extracted from the deconvoluted spectrum.

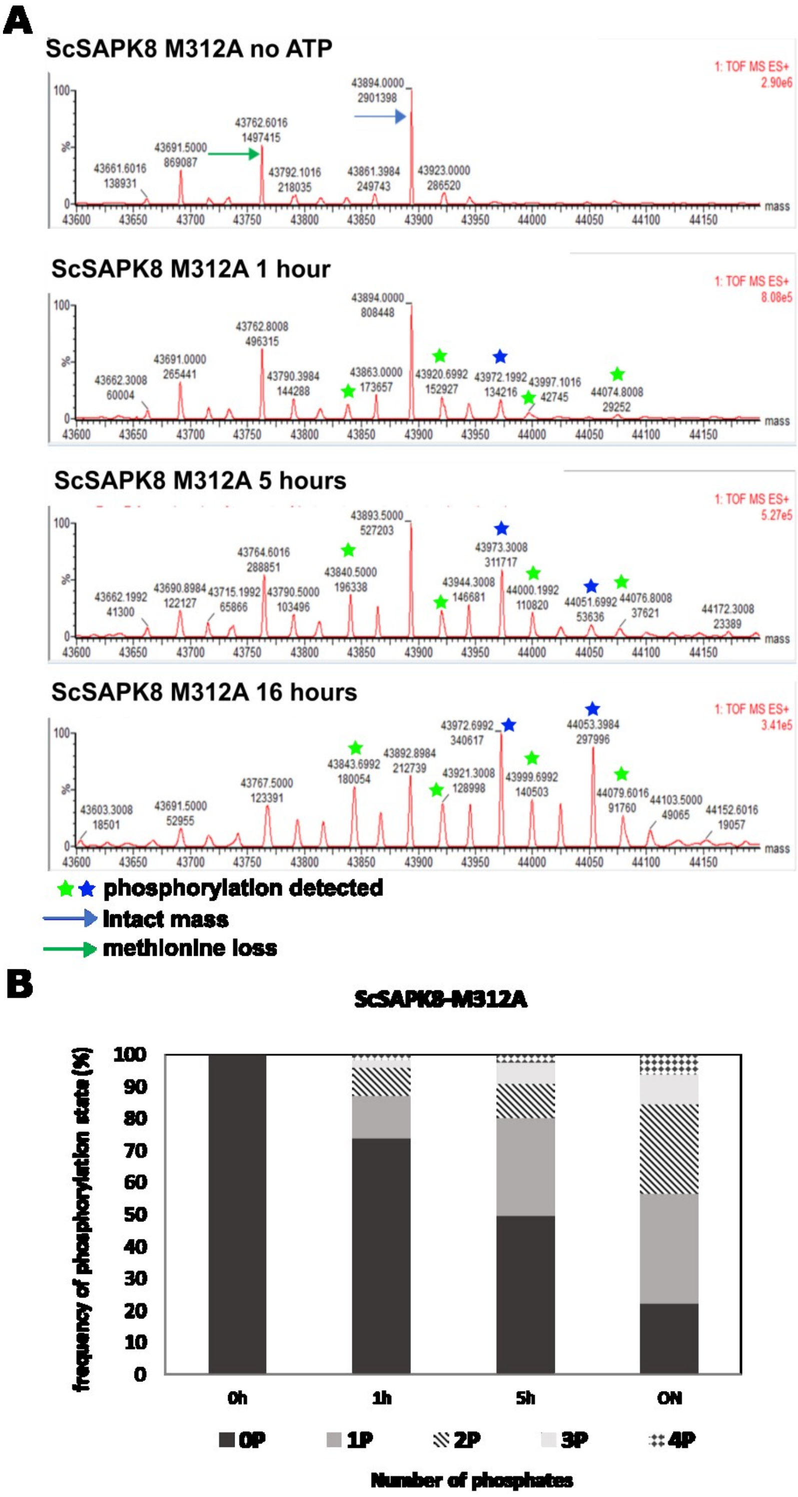

Supplementary Figure S6: ScSAPK8-M312A autophosphorylation. A: Deconvoluted mass spectrum at time 0, 1 hour, 5 hours and overnight, generated by MAX ENT1 software (Waters). The blue arrow represents the intact kinase mass, and the blue stars represent the detected masses with one or more phosphorylations. The green arrows indicate the exact kinase mass with the loss of methionine that could occur during protein expression. The green stars represent masses of this kinase form with one or more phosphorylations. **B:** Graphical representation of protein autophosphorylation over time. The percentage values were calculated using the ion counts extracted from the deconvoluted spectrum.

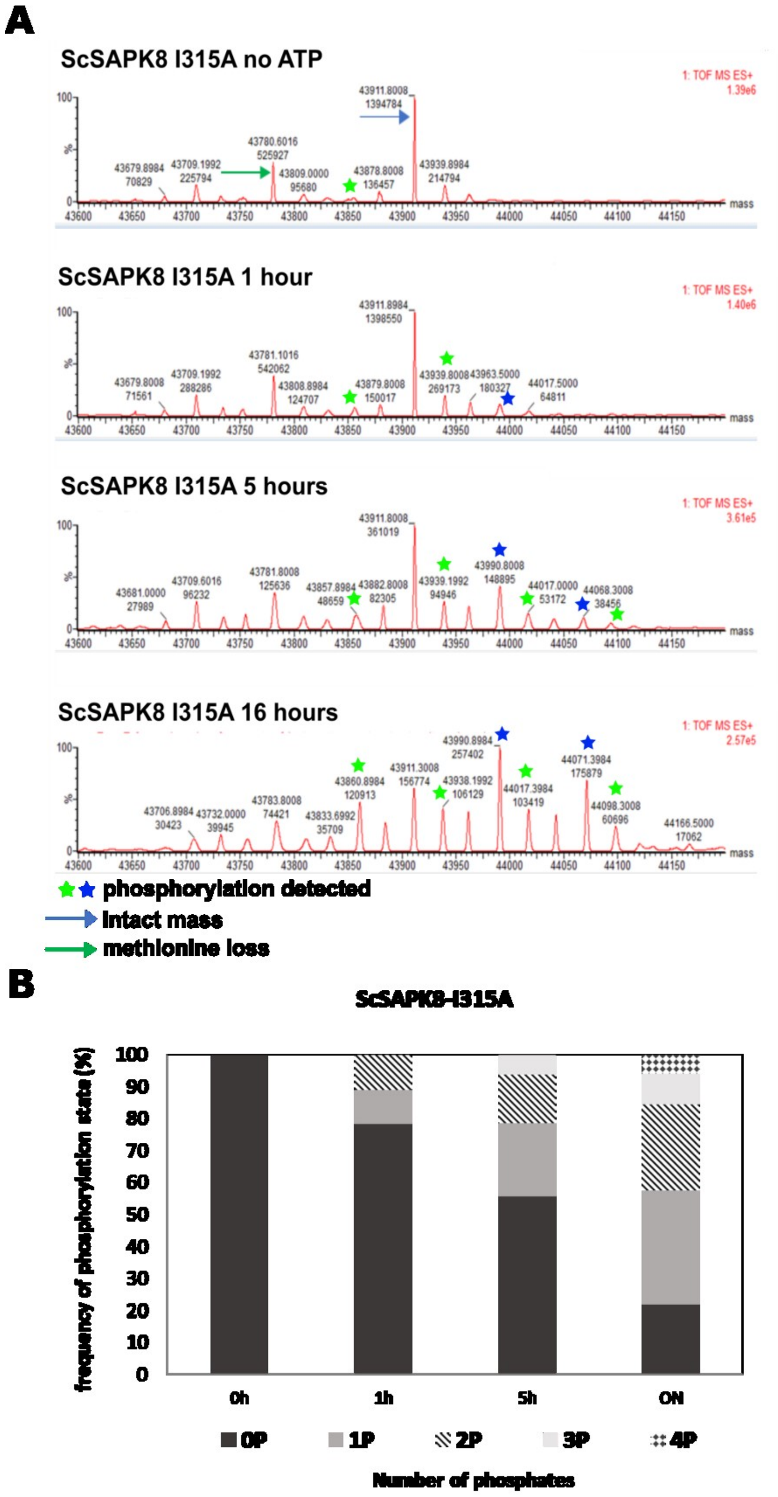

Supplementary Figure S7: ScSAPK8-I315A autophosphorylation. A: Deconvoluted mass spectrum at time 0, 1 hour, 5 hours and overnight, generated by MAX ENT1 software (Waters). The blue arrow represents the intact kinase mass, and the blue stars represent the detected masses with one or more phosphorylations. The green arrows indicate the exact kinase mass with the loss of methionine that could occur during protein expression. The green stars represent masses of this kinase form with one or more phosphorylations. **B:** Graphical representation of protein autophosphorylation over time. The percentage values were calculated using the ion counts extracted from the deconvoluted spectrum.

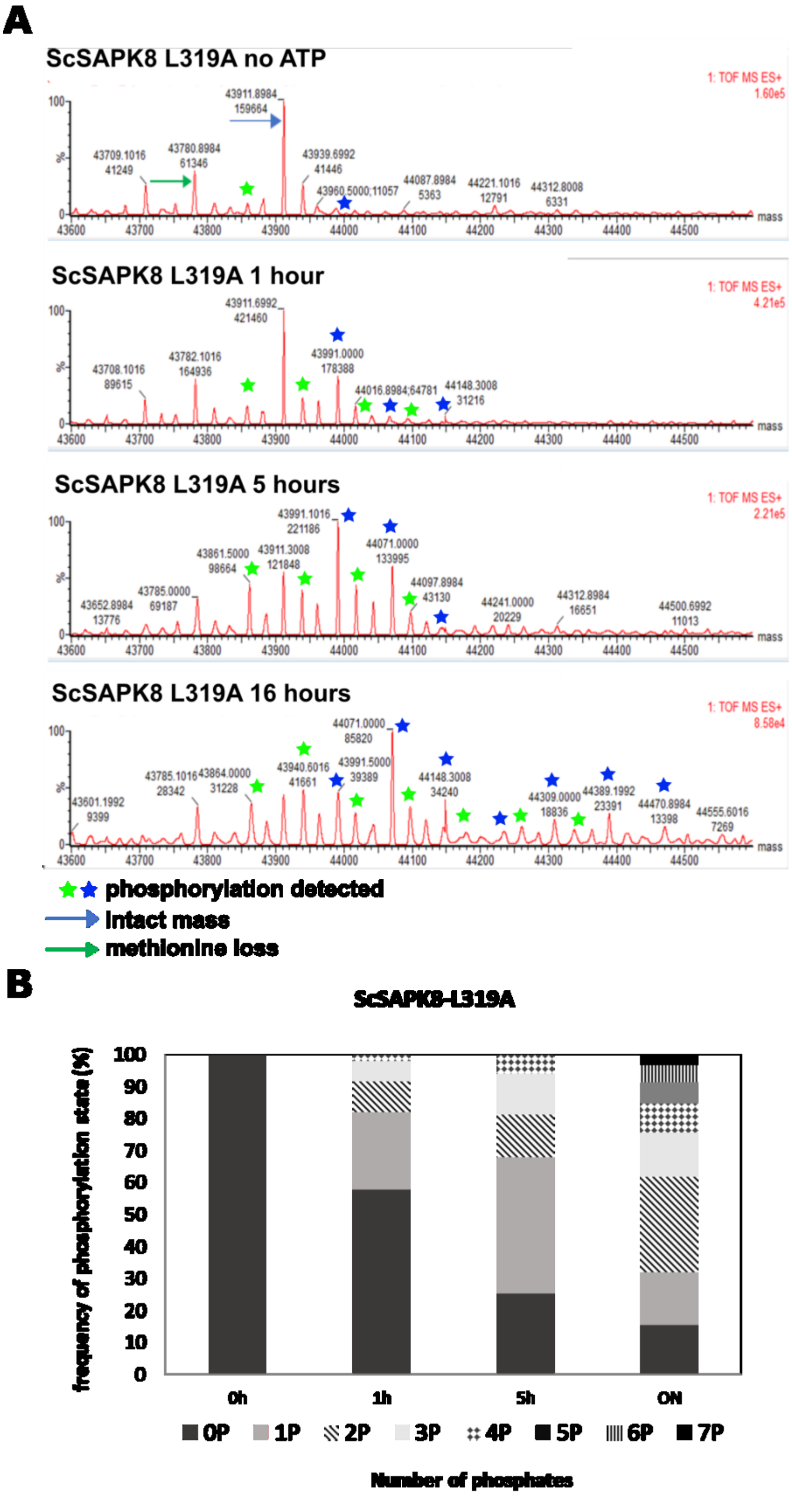

Supplementary Figure S8: ScSAPK8-L319A autophosphorylation. A: Deconvoluted mass spectrum at time 0, 1 hour, 5 hours and overnight, generated by MAX ENT1 software (Waters). The blue arrow represents the intact kinase mass, and the blue stars represent the detected masses with one or more phosphorylations. The green arrows indicate the exact kinase mass with the loss of methionine that could occur during protein expression. The green stars represent masses of this kinase form with one or more phosphorylations. **B:** Graphical representation of protein autophosphorylation over time. The percentage values were calculated using the ion counts extracted from the deconvoluted spectrum.

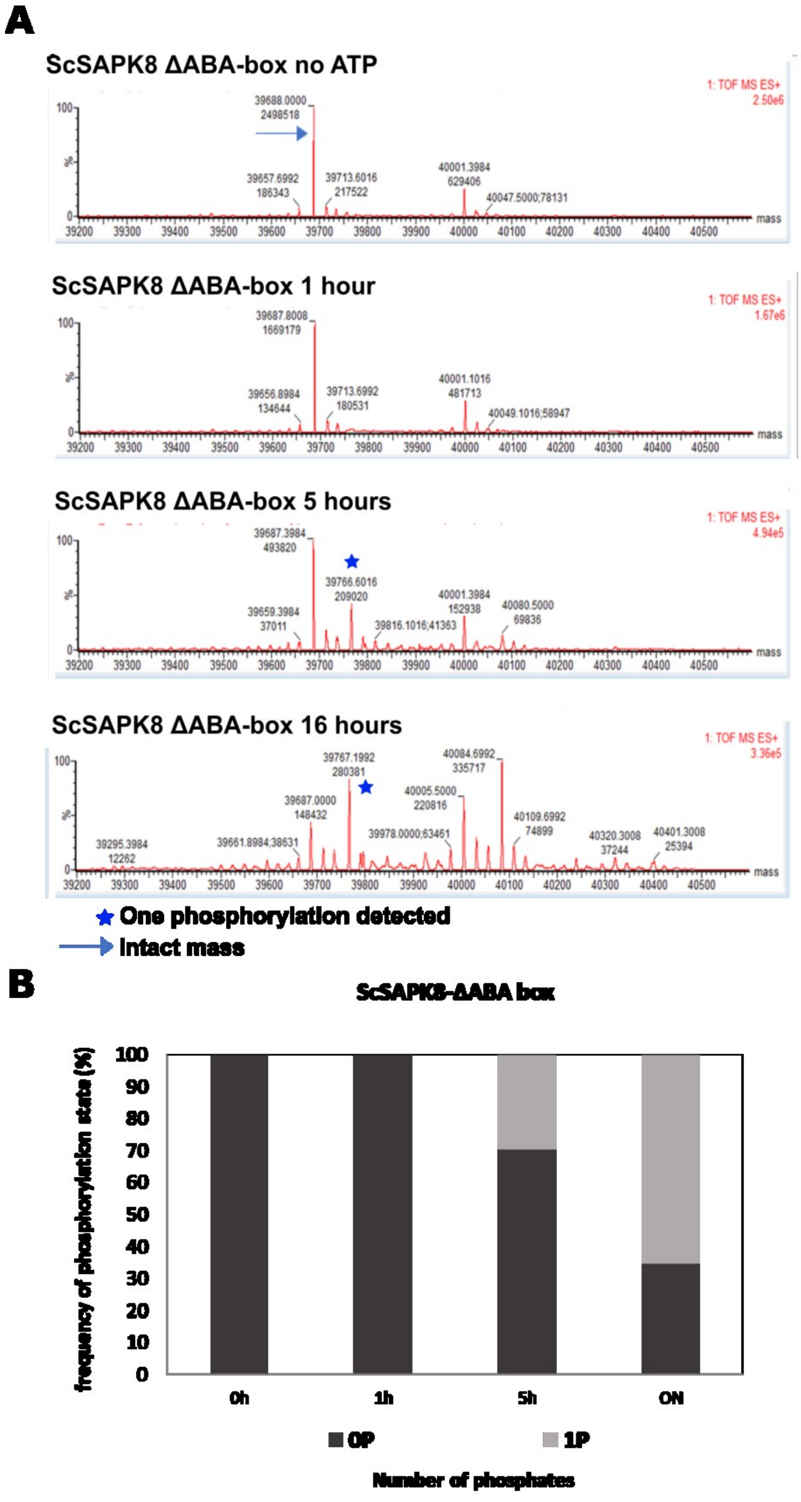

Supplementary Figure S9: ScSAPK8-ΔABA-box autophosphorylation. A: Deconvoluted mass spectrum at time 0, 1 hour, 5 hours and overnight, generated by MAX ENT1 software (Waters). The blue arrow represents the intact kinase mass, and the blue stars represent the detected masses with one or more phosphorylations. **B:** Graphical representation of protein autophosphorylation over time. The percentage values were calculated the ion counts extracted from the deconvoluted spectrum.

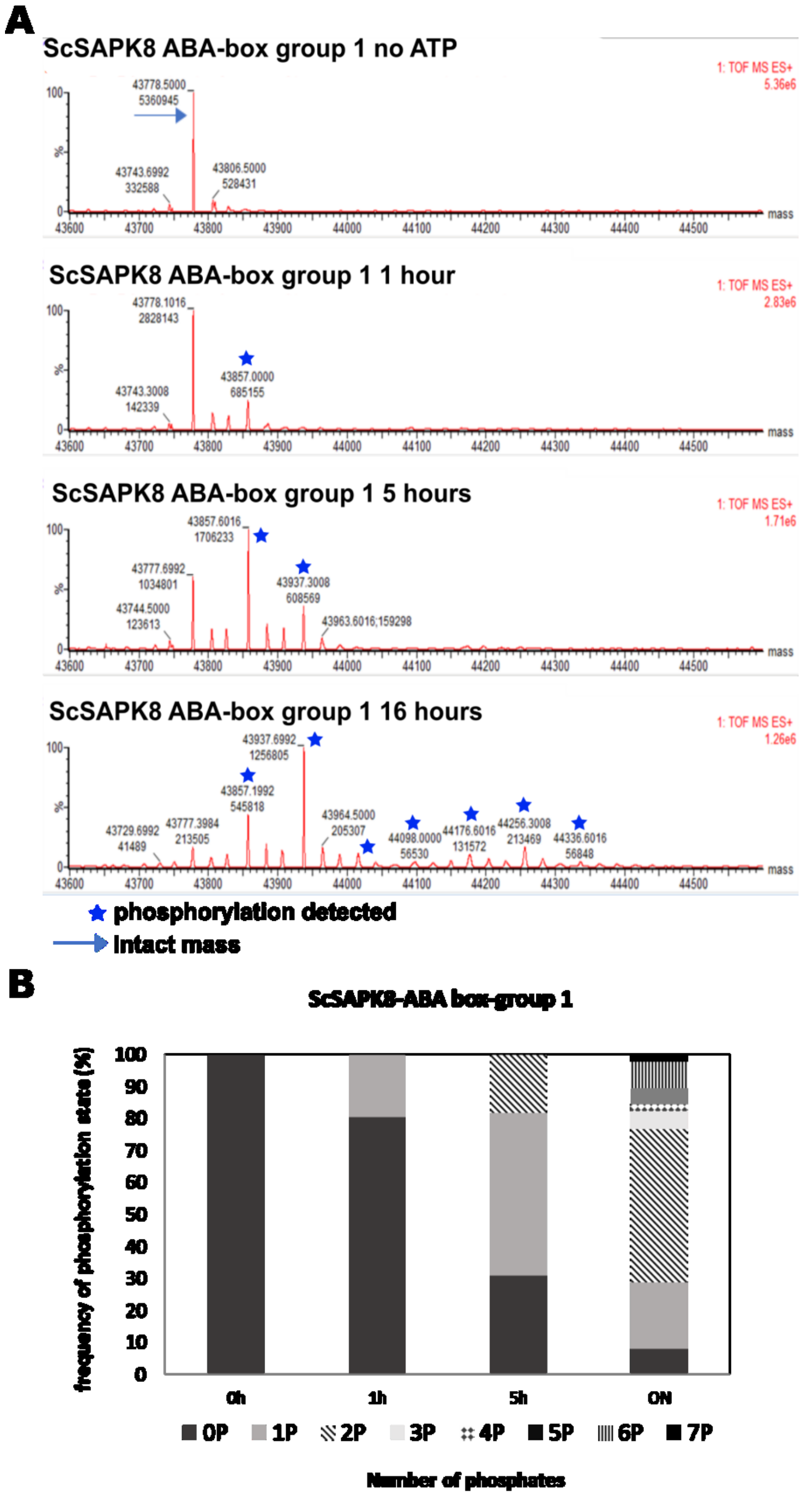

Supplementary Figure S10: ScSAPK8-ABAbox-group1 autophosphorylation. A: Deconvoluted mass spectrum at time 0, 1 hour, 5 hours and overnight, generated by MAX ENT1 software (Waters). The blue arrow represents the intact kinase mass, and the blue stars represent the detected masses with one or more phosphorylations. **B:** Graphical representation of protein autophosphorylation over time. The percentage values were calculated the ion counts extracted from the deconvoluted spectrum.

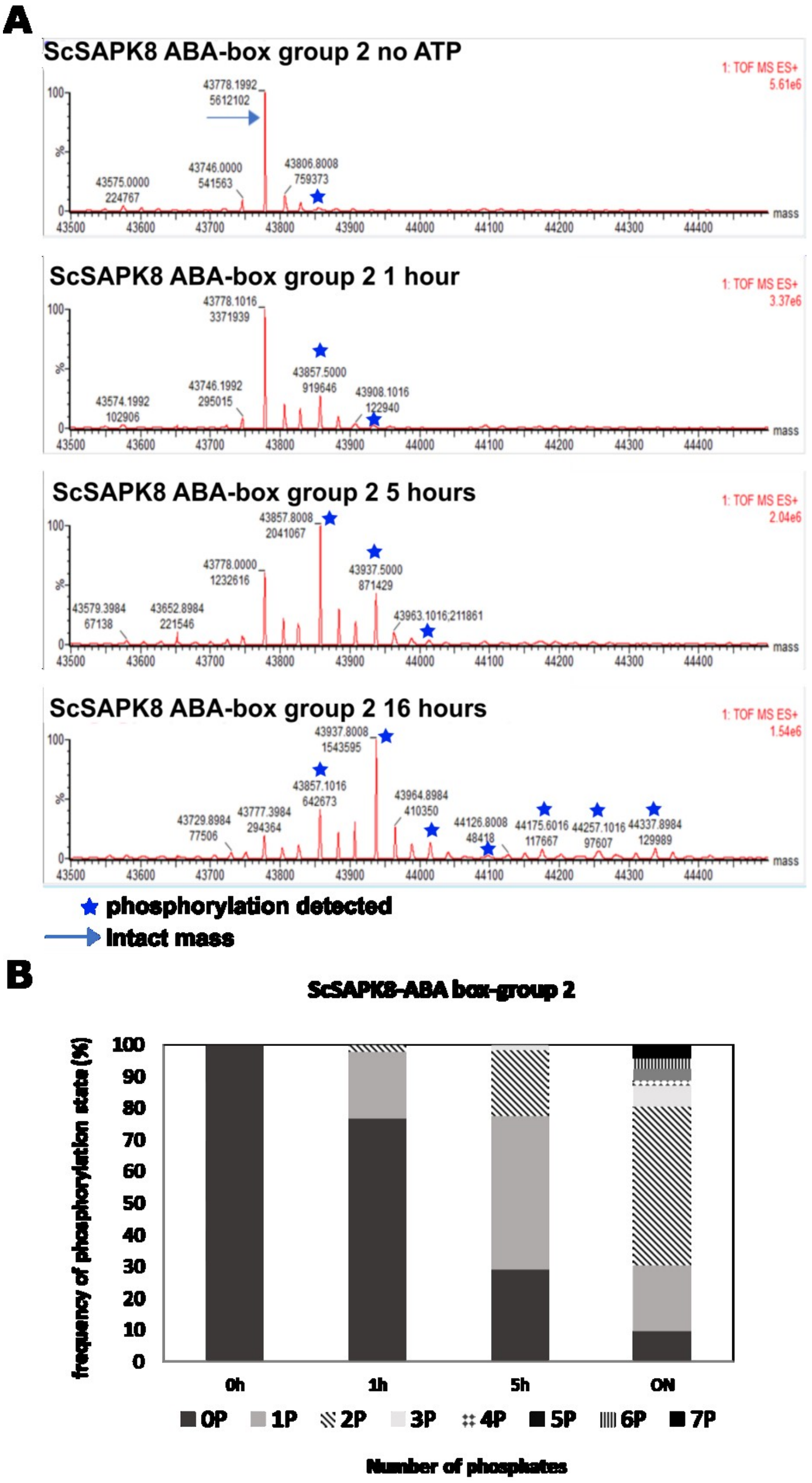

Supplementary Figure S11: ScSAPK8-ABAbox-group2 autophosphorylation. A: Deconvoluted mass spectrum at time 0, 1 hour, 5 hours and overnight, generated by MAX ENT1 software (Waters). The blue arrow represents the intact kinase mass, and the blue stars represent the detected masses with one or more phosphorylations. **B:** Graphical representation of protein autophosphorylation over time. The percentage values were calculated the ion counts extracted from the deconvoluted spectrum.

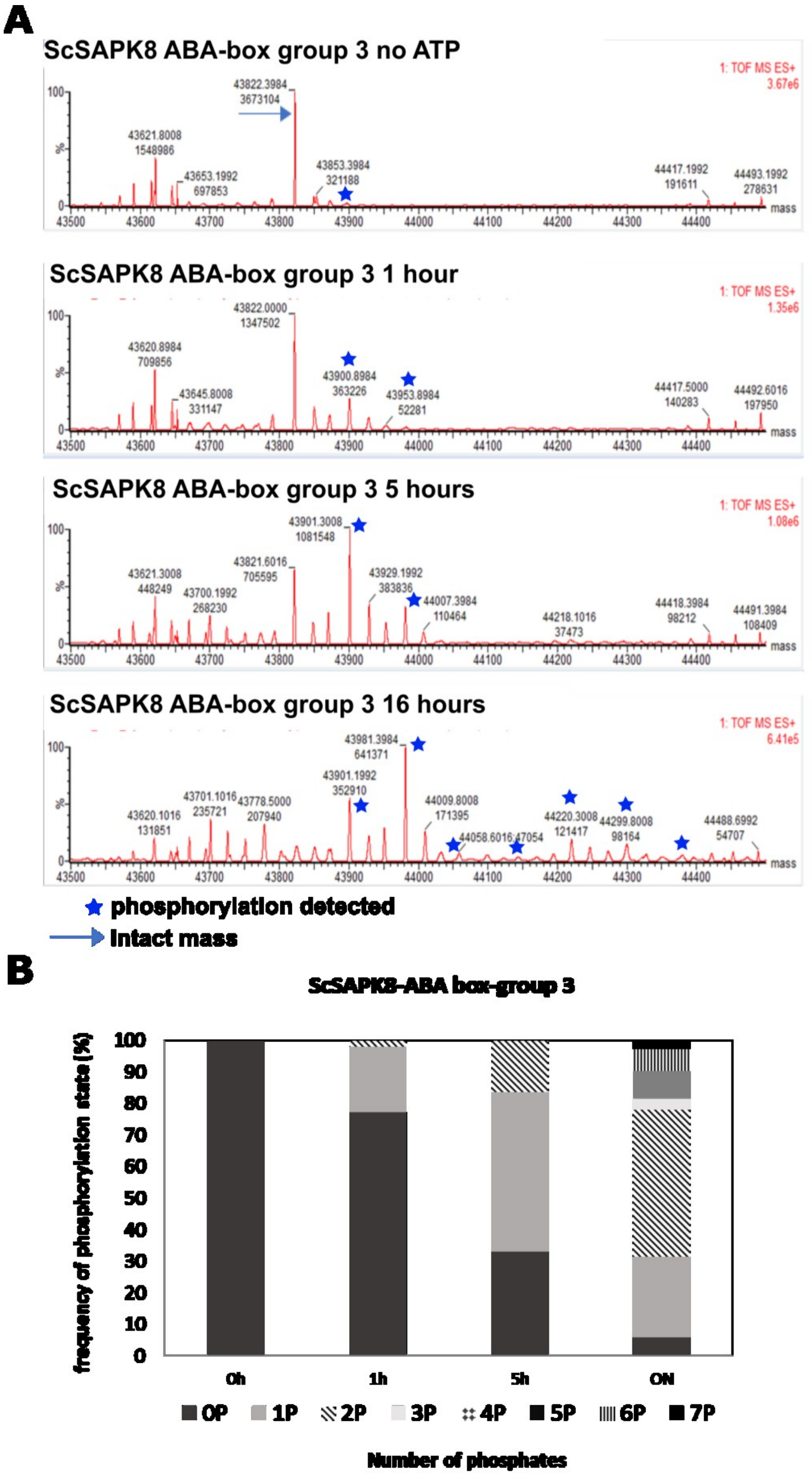

Supplementary Figure S12: ScSAPK8-ABAbox-group3 autophosphorylation. A: Deconvoluted mass spectrum at time 0, 1 hour, 5 hours and overnight, generated by MAX ENT1 software (Waters). The blue arrow represents the intact kinase mass, and the blue stars represent the detected masses with one or more phosphorylations. **B:** Graphical representation of protein autophosphorylation over time. The percentage values were calculated the ion counts extracted from the deconvoluted spectrum.

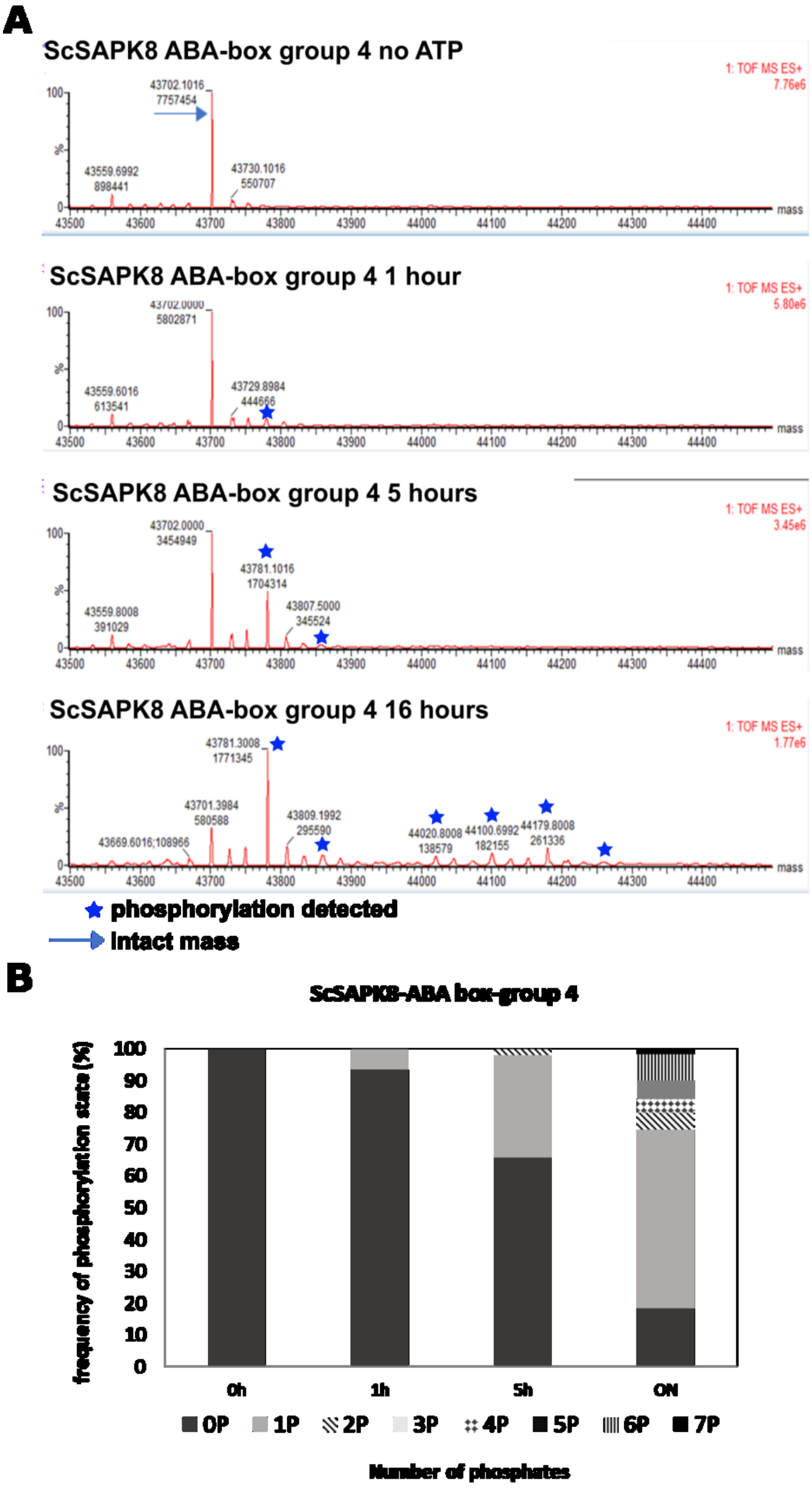

Supplementary Figure S13: ScSAPK8-ABAbox-group4 autophosphorylation. A: Deconvoluted mass spectrum at time 0, 1 hour, 5 hours and overnight, generated by MAX ENT1 software (Waters). The blue arrow represents the intact kinase mass, and the blue stars represent the detected masses with one or more phosphorylations. **B:** Graphical representation of protein autophosphorylation over time. The percentage values were calculated the ion counts extracted from the deconvoluted spectrum.

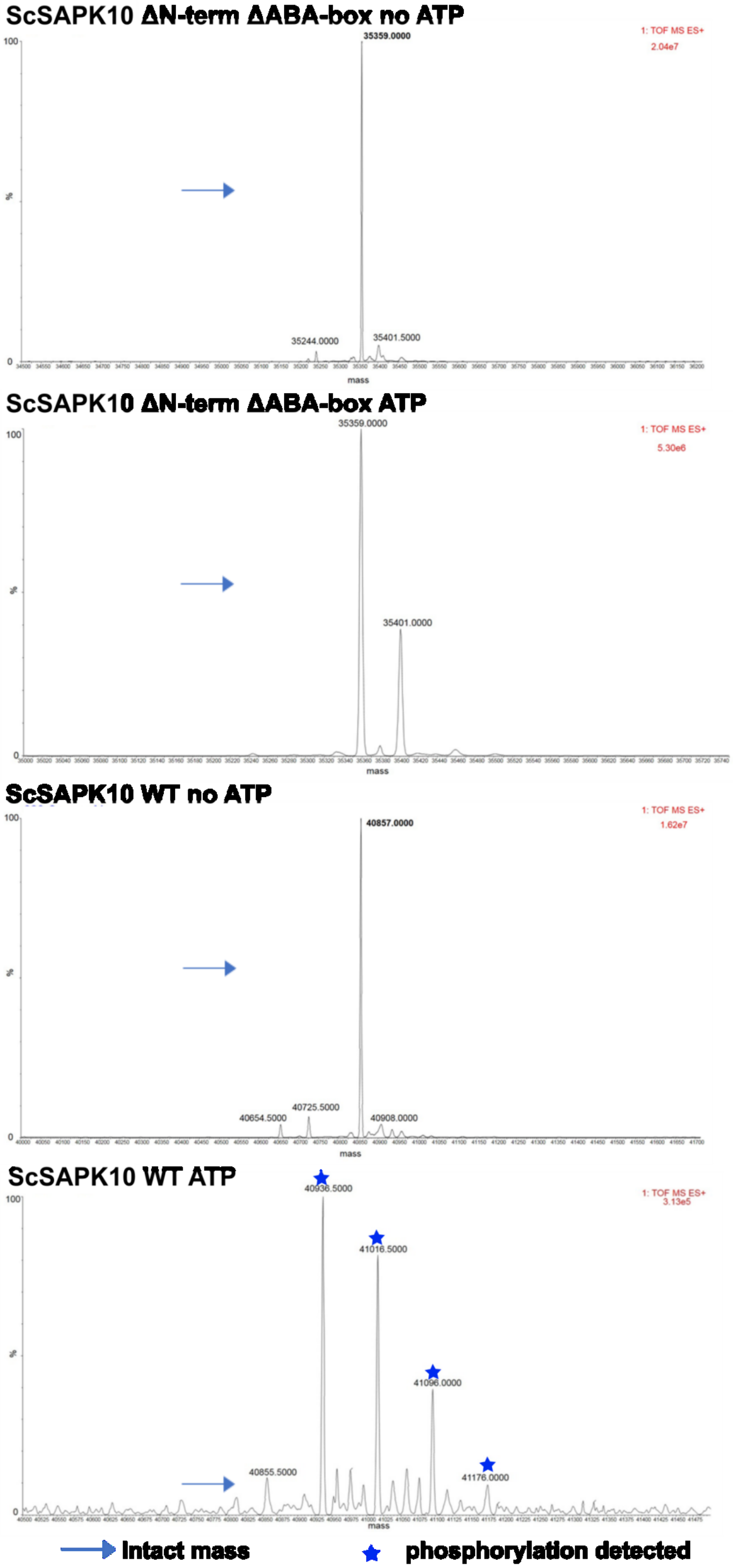
ScSAPK10 ΔN-term ΔABA-box no ATP
35359.0000
35244.0000
35401.5000
1: TOF MS ES+
2.04e7
mass
ScSAPK10 ΔN-term ΔABA-box ATP
35359.0000
35401.0000
1: TOF MS ES+
5.30e6
mass
ScSAPK10 WT no ATP
40857.0000
40654.5000
40725.5000
40908.0000
1: TOF MS ES+
1.62e7
mass
ScSAPK10 WT ATP
40936.5000
41016.5000
41096.0000
41176.0000
40855.5000
1: TOF MS ES+
3.13e5
mass
Intact mass
phosphorylation detected

Supplementary Figure S14: ScSAPK10 WT and mutant ScSAPK10 ΔN-term ΔABA-box autophosphorylation. Deconvoluted mass spectrum at time 1 hour generated by MAX ENT1 software (Waters). The blue arrow represents the intact kinase mass, and the blue stars represent the detected masses with one or more phosphorylations.

Table S1: List of primer sequences

Primer Name	Sequence 5' - 3'	Used for
Sc_SAPK8_MluI_NdeI_F	ACGCGTCATATGATGGCAGGGCCGGCGCCG	isolation from cDNA and cloning
Sc_SAPK8_NotI_R	GCGGCCGCCATTGCGTACACAATCTCACC	
Sc_SAPK9_MluI_NdeI_F	ACGCGTCATATGATGGCGAGGACGCCGGCA	
Sc_SAPK9_NotI_R	GCGGCCGCCATGGCATACACTATCTCTCC	
Sc_SAPK10_Mlu_NdeI_F	ACGCGTCATATG ATGGACCGGGCGGCGCTC	
Sc_SAPK10_NotI_R	GCGGCCGCCATAGCATACACGATCTCCC	
SAPK8_full_S	TACTTCCAATCCATGGCAGGGCCGGCG	cloning in pNIC28-Bsa4 vector
SAPK8_full_AS	TATCCACCTTTACTGTCACATTGCGTACACAATCTCACC	
SAPK9_full_S	TACTTCCAATCCATGGCGAGGACGCCG	
SAPK9_full_AS	TATCCACCTTTACTGTCACATGGCATACACTATCTCTCC	
SAPK10_full_S	TACTTCCAATCCATGGACCGGGCGGCG	
SAPK10_full_AS	TATCCACCTTTACTGTCACATAGCATACACGATCTCCCC	
SAPK10_dm_S	TACTTCCAATCCATGGACATGCCCATAATGCAC	ScSAPK10 truncation
SAPK10_dm_AS	TATCCACCTTTACTGTCATGGAATGGTCGCCTCGG	
ScSAPK8_M312A_S	AATGCAGACCGCGGATCAGATCA	SnRK2-box site-directed mutagenesis
ScSAPK8_M312A_AS	TGATCTGATCCGCGGTCTGCATT	
ScSAPK8_I315A_S	ATGGATCAGGCCATGCAGATTTTG	
ScSAPK8_I315A_AS	CAAAATCTGCATGGCCTGATCCATGG	
ScSAPK8_L319A_S	CATGCAGATTGCGACAGAGGCCA	
ScSAPK8_L319A_AS	TGGCCTCTGTCGCAATCTGCATG	
ScSAPK8_group1_S	GATGGATTGGCCATGGCCGCCGCCATGGATGAT	ABA-box site-directed mutagenesis
ScSAPK8_group1_AS	ATCATCCATGGCGGCGGCCATGGCCAATCCATC	
ScSAPK8_group2_S	CGACGACATGGCTGCTCTTGCCTCCGCCTCAGATCTTG	
ScSAPK8_group2_AS	CAAGATCTGAGGCGGAGGCAAGAGCAGCCATGTCGTCG	
ScSAPK8_group3_S	TCCGACTCAGCTCTTGCTGTTGCCAGCAGCGGT	
ScSAPK8_group3_AS	ACCGCTGCTGGCAACAGCAAGAGCTGAGTCGGA	
ScSAPK8_group4_S	AGCAGTGGAGCGGCTGTGGCCGCAGCGTGACAGTAAAGGTGGATA	
ScSAPK8_group4_AS	TATCCACCTTTACTGTCACGCTGCGGCCACAGCCGCTCCACTGCT	
SAPK8_full_S	TACTTCCAATCCATGGCAGGGCCGGCG	ABA-box deletion
SAPK8_dm_AS	TATCCACCTTTACTGTCAAGGTGGTATGGTGGCCTC	
